# Internet of things enabled smart agriculture: Current status, latest advancements, challenges and countermeasures

**DOI:** 10.1016/j.heliyon.2025.e42136

**Published:** 2025-01-22

**Authors:** Navod Neranjan Thilakarathne, Muhammad Saifullah Abu Bakar, Pg Emeroylariffion Abas, Hayati Yassin

**Affiliations:** Faculty of Integrated Technologies, Universiti Brunei Darussalam, Gadong, BE1410, Brunei

**Keywords:** Agriculture 4.0, Artificial intelligence, Internet of things, IoT, Precision agriculture, Robotics, Smart agriculture, Smart farming, Sustainability

## Abstract

It is no wonder that agriculture plays a vital role in the development of some countries when their economies rely on agricultural activities and the production of food for human survival. Owing to the ever-increasing world population, estimated at 7.9 billion in 2022, feeding this number of people has become a concern due to the current rate of agricultural food production subjected to various reasons. The advent of the Internet of Things (IoT) based technologies in the 21st century has led to the reshaping of every industry, including agriculture, and has paved the way for smart agriculture, with the technology used towards automating and controlling most aspects of traditional agriculture. Smart agriculture, interchangeably known as smart farming, utilizes IoT and related enabling technologies such as cloud computing, artificial intelligence, and big data in agriculture and offers the potential to enhance agricultural operations by automating and making intelligent decisions, resulting in increased efficiency and a better yield with minimum waste. Consequently, most governments are spending more money and offering incentives to switch from traditional to smart agriculture. Nonetheless, the COVID-19 global pandemic served as a catalyst for change in the agriculture industry, driving a shift toward greater reliance on technology over traditional labor for agricultural tasks. In this regard, this research aims to synthesize the current knowledge of smart agriculture, highlighting its current status, main components, latest application areas, advanced agricultural practices, hardware and software used, success stores, potential challenges, and countermeasures to them, and future trends, for the growth of the industry as well as a reference to future research.

## 1 Introduction

Despite the low impression that many people have towards agriculture as a profession, the reality is that continuous agricultural food production is essential for human survival [1,2]. Throughout the course of history, humans have relied on agricultural food production after giving up hunting and nomadic lifestyles 12000 years ago [1,3]. Traditional hunting lifestyles were abandoned in favour of permanent communities and consistent food sources, and cities and civilizations emerged as a result of agriculture [2]. With agriculture allowing the production of food and animals according to demand, the global population has subsequently exploded from about five million people to over seven billion in 10,000 years [1]. In diverse places of the world, people started farming, as a source of their main livelihood [2,3], making agriculture the primary and oldest industry in the world. Over the years, the agriculture industry has evolved but still remains a crucial industry towards economic development and balancing social stability [4,5].

**Fig. 1:**
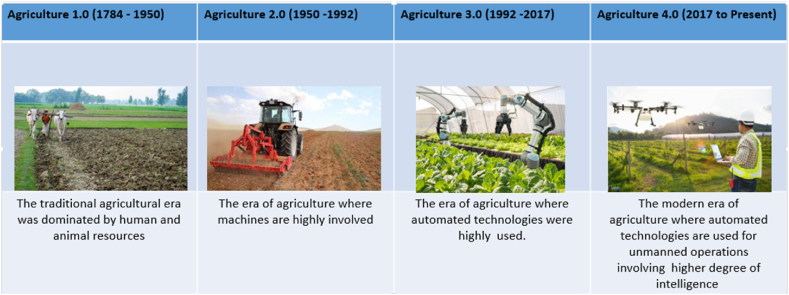
Evolution of Agriculture (from the era of Agriculture 1.0 to Agriculture 4.0).

From generation to generation, the traditional approach of agriculture has undergone many transformations and has progressively evolved, aiming to improve overall productivity while confronting a variety of challenges [[4], [5], [6], [7]]. Along the historical timeline, the transformation of agriculture can be categorized into several stages [[1], [2], [3], [4], [5]]; Agriculture 1.0, 2.0, 3.0, and afterward 4.0, as depicted in Fig. 1.

• Agriculture 1.0 (Pre-Industrial Era): The traditional agricultural era (1784–1870) relied heavily on human and animal labor. Tools like the wooden plow and irrigation canals represented early attempts to improve efficiency. However, these methods were labor-intensive and slow, leading to limited productivity and significant operational inefficiencies [[1], [2], [3], [4]].
• Transition to Agriculture 2.0: The inefficiencies of manual labor and the growing need to cultivate larger areas led to the introduction of mechanized tools during the Industrial Revolution [[1], [2], [3], [4]]. Steam powered engines and mechanical threshers reduced reliance on animal labor, enabling larger-scale farming.
• Agriculture 2.0 (Industrial Mechanization): In the 20th century, man-made machinery revolutionized agricultural practices, significantly boosting productivity. However, the mechanization of agriculture also introduced challenges such as resource inefficiency and environmental degradation due to the indiscriminate use of machinery. These inefficiencies became a fundamental issue during the period from 1950 to 1992 [[1], [2], [3]].
• Transition to Agriculture 3.0: To address resource inefficiency and labor shortages, the mid 20th century saw the development of automated systems like robotic controls and early irrigation controls. These advancements allowed for better resource management but still lacked data-driven intelligence.
• Agriculture 3.0 (Automation): Between 1992 and 2017, agricultural processes saw rapid advancements in automation, which improved consistency and reduced manual effort. However, a significant limitation during this period was the reliance on intuition rather than data-driven decision-making, resulting in a low degree of operational intelligence [1,4].
• Transition to Agriculture 4.0: The digital revolution introduced IoT, AI, and big data, enabling real-time monitoring and precision farming. These technologies addressed the need for data-driven decisions, enhancing productivity, and sustainability.
• Agriculture 4.0 (Smart Farming): The smart agricultural era, which began in 2017, is defined by autonomous operations powered by advanced information and communication technologies [1,4]. By leveraging interconnected devices and sophisticated analytics, smart agriculture optimizes every aspect of farming, from planting to harvesting; enhancing efficiency, sustainability, and adaptability to climate challenges.

In the current era of Agriculture 4.0, also known as Data-Driven Agriculture [8,9], agricultural activities are deeply integrated with modern information technologies governed by IoT, big data, cloud computing, and artificial intelligence (AI), to execute agricultural tasks more efficiently and effectively [[6], [7], [8], [9]]. In short, smart agriculture combines agriculture with IoT systems to create opportunities for the development of agricultural production and utilize fewer resources [4,[10], [11], [12], [13], [14]]. Even though many researchers have stated that we are currently in the era of Agriculture 4.0, a few researchers have stated that as of now [[14], [15], [16]], we are currently at the dawn of Agriculture 5.0, which focuses more on IoT, AI, unmanned machinery, and big data to increase agricultural operations as opposed to agriculture 4.0. Not only that but also, they emphasized that apart from these technical breakthroughs, Agriculture 5.0 will also combine green energy sources to increase the efficiency of the agriculture sector. The availability of renewable energy sources such as wind, solar, and biomass energy is not restricted, which means combining green energy sources, such as renewable energy sources, with emerging technology in agriculture can be extremely lucrative for the industry. Therefore, farmers that use this form of energy may have a long-term source of income while also attaining a reduction in their environmental imprint and becoming more effective in the approaching era of Agriculture 5.0.

According to the United Nations Food and Agriculture Organization [14], the world population is expected to reach 10 billion by 2050 and may reach 11 billion by the end of the century [12,14]. This places a huge demand on the agricultural sector, necessitating the expansion of agricultural food production to feed the growing population. As a result, it may be necessary to increase agricultural production by 70 % by 2050 to feed this number of people [11,12,14]. Cities, on the other hand, have a higher population density than rural areas. As the population grows, this disparity is expected to widen, resulting in a loss of arable land and water supplies. According to the studies [6,7], total arable land for food production was estimated to be around 20 million square miles (40 % of the world's land) in 2013 and eventually dropped to 18 million square miles (38 % of the world's land area) as a result of rapid urbanization, resulting in a food supply-demand gap.

**Fig. 2:**
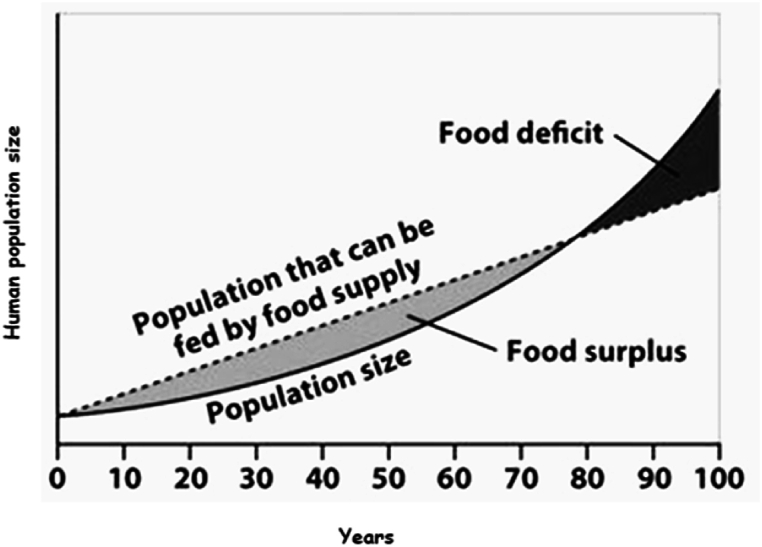
Population growth and demand for food supply [14,[18], [19], [20]].

In addition, as the global population ages, there will be fewer people available to work, especially in rural regions of low-income nations that rely mostly on agricultural output for their economy [[14], [15], [16], [17], [18]]. Farmers also prefer traditional farming methods because they are hesitant to use new technologies [12]. Overall agricultural output may be reduced due to declining arable land owing to urbanization, aging, and farmer's reluctance to adopt new technologies. Furthermore, climate change challenges, such as the increased occurrence of natural disasters, for example, droughts, floods, and transboundary pests and diseases, are reducing agricultural output. Climate change does, in fact, have a disproportionately negative impact on food-insecure areas, threatening crop and livestock production [16]. Taking all of this into account, meeting rising agricultural demand with current farming practices will almost certainly result in increased competition for natural resources, increased greenhouse gas emissions, and further deforestation and land degradation, ultimately leading to food shortages and worsening the situation [14]. Fig. 2 depicts how food shortages would develop over time, in tandem with the world's ever-increasing population [14,[18], [19], [20]]. According to Fig. 2, there was a surplus of food supply in the early years due to the size of the world population, whereas as time passed due to the exponential growth of the world population and the slow growth rate of food production, there would be a food deficit, which would eventually become an agony if not managed beforehand.

Expansion of agricultural food production and economic progress have frequently come at a cost to the environment, and it always will be the same unless there is a way to utilize available resources with maximum efficiency [12]. Nearly half of the world's forests have already vanished, and the availability of groundwater is rapidly diminishing [3]. Biodiversity has suffered significantly, and annually, billions of tons of greenhouse gases are released into the atmosphere as a result of the combustion of fossil fuels, causing global warming and climate change [14]. All of these negative trends are intensifying, and agriculture is a significant contributor to the situation [[20], [21], [22], [23], [24]]. Deforestation, primarily for agricultural purposes, contributes significantly to global greenhouse gas emissions and results in habitat damage, species extinction, and biodiversity degradation [14]. These trends jeopardize the long-term viability of agricultural systems and endanger the world's ability to meet its food requirements, raising doubt on environmental sustainability. In return, even little changes in the environment, such as variations in yearly rainfall or seasonal precipitation patterns, can have a significant impact on agricultural food production as well [25,26].

The aforementioned issues have been recognized as critical by the world community and according to the 2030 Agenda for Sustainable Development Goals, which was endorsed by the United Nations in September 2015, offers a compelling yet challenging vision of how many objectives might be merged to define new sustainable development paths towards solving such critical issues before they become worsen. As per the United Nations plan, the second sustainable development goal aims to end hunger, increase food security and nutrition, and promote sustainable agriculture by 2030 [11,12]. Looking forward, the main challenge is whether today's agriculture systems will be able to meet the needs of a worldwide population that is expected to reach more than 10 billion people by 2050, where the current production must be boosted by more than 70 % [12]. The promising question is whether we will be able to meet the required output or reach the expectations as there are constraints on already scarce land and water resources, as well as the rising adverse effects of climate change [14,[26], [27], [28], [29], [30]]. The answer is that current agricultural systems are capable of providing enough food without any shortages but doing so in a comprehensive and sustainable manner will necessitate considerable change, which requires the adoption of new technology, smart agriculture.

Smart agriculture employs a number of technologies, the backbone of which is IoT [1,2], at various steps to improve agricultural production, reducing the cost by minimizing the use of resources such as fertilizer, pesticides, fuel, and manual labour, allowing farmers to overcome the aforementioned challenges pertaining to the traditional agriculture with minimum efforts [10], while combining higher intelligence [[31], [32], [33], [34]]. For instance, in smart agriculture, various types of sensors can be used to gather environmental data (e.g., humidity, temperature, pressure, light intensively, etc.) for irrigation [[3], [4], [5], [6], [7],^27^], plant and crop monitoring [[3], [4], [5], [6], [7],^24^], soil condition [[3], [4], [5], [6], [7],^23^,^27^], pest control [[3], [4], [5], [6]], disease diagnosis [[3], [4], [5], [6]], and delivery tracking [[3], [4], [5], [6], [7]]; and then communication networks to receive and send data using a variety of communication protocols, which then analyzed and managed by data analysis solutions and decision support systems. Such sensors used in smart agriculture include sensors that can sense humidity, temperature, pressure, luminosity, Global Positioning System (GPS), and ground chemical concentration [3,[5], [6], [7], [8],[10], [11], [12], [13], [14]]. This system of interconnected devices is commonly referred to as IoT, which gathers data to boost agricultural productivity and eliminate waste by taking intelligent decisions [10,12]. In a traditional farming setting, farmers must regularly visit the farming fields thorough out the crop life cycle to have a better sense of crop conditions, whereas the adoption of smart agriculture allows up to 80 % of the time to be spent on monitoring and understanding the state of the crops rather than regularly visiting and taking care of crops in the field.

Currently, the global market for smart agriculture is predicted to rise from 9.58 billion US dollars in 2017 to over 40 billion US dollars by 2030 [12,[15], [16], [17]]; and it is predicted that it will continue to rise with a higher margin over the next few years due to the rising demand, which signifies its importance. Since its onset in December 2019, the COVID-19 pandemic had a profound impact globally, heavily challenging the economies of most countries during its course [[15], [16], [17], [18]]. Subsequently, agricultural production and the behaviour and patterns are heavily changed, and owing to the contagious nature of the COVID-19 virus, the health of farmers, supply chain interruptions, and labour shortages have become significant issues in terms of agricultural production [[18], [19], [20]], resulting in food shortages and inflation (e.g., food shortage and record-high inflation of Sri Lanka from 2021 to until now, which started with the emergence of pandemic aided by the policy changes of the government to ban fertiliser). These concerns posed during the pandemic and post-pandemic period have led countries to emphasize on introducing automated technologies to the production system. As a result, specific efforts are essential to improve the food supply system and prepare for future emergencies as the onset of the next global disaster cannot be guaranteed. The necessity of being able to conduct agricultural chores from a distance has been highlighted by the current outbreak [12]. As a result, smart agricultural solutions are expected to drive future market and can assist farmers in recovering from losses in significantly less time [12,14], as well as enabling farmers to maximize food production by optimizing various farming conditions, such as improving fertilizer distribution to the soil, minimizing pesticide use, and reducing irrigation water usage [19]. Thus, motivated by the prospect of uncovering the most recent applications, practices, software, and hardware already in use, as well as the challenges and how to overcome such challenges pertaining to IoT-aided smart agriculture, the primary motivators for conducting this research are mentioned below.

• Even though smart agriculture is a field that is extensively studied, owing to the ever-changing nature of modern technologies (e.g., IoT), the field is getting updated day by day, creating new avenues for novel research and innovations.
• It is evident that less research work has been carried out in terms of how COVID-19 affects current smart agriculture practices, as the entire world has suffering through this pandemic for nearly four years, which has impacted many industries, including agriculture, making this a crucial topic worth scrutinizing.
• As the world population grows, more and more people will migrate to cities anticipating more future prospects resulting in higher urbanization, where the side effect would be the depletion of arable land and decline of water sources. It has been noted that less research has been carried out focusing on how smart agriculture can be a remedy for this urbanization. On the other hand, even though there are already existing surveys/reviews available pertaining to smart agriculture none of them provide views on how the countries have thrived with the adoption of smart agriculture, and even though they have highlighted potential challenges, none of them discussed what countermeasures can be taken. Thus, all these factors have motivated us to find answers and make a comprehensive review.

### 1.1 Contributions of the study

As above mentioned, motivated by the fact that synthesizing the latest knowledge pertains to smart agriculture, we aim to provide an overview of the present practice of smart agriculture, highlighting how it is made out from, the newest application areas, advanced agricultural practices, success stories, latest software and hardware that are being utilized, a summary of existing work and the latest research, key challenges and countermeasures, and future directions. Hence the key contributions of the study are outlined as follows.

• Following the introduction, a preamble on IoT in smart agriculture has been provided highlighting its main components; as being the core backbone of smart agriculture.
• Key application areas of smart agriculture are highlighted with their current status and recent developments. Further, the latest agricultural practices are also highlighted.
• To open the way for future research, we highlight the success stories of some countries that now excel well in smart agriculture and provide a brief overview of the latest software and hardware currently being utilized in smart agriculture.
• To differentiate our work from others, we have provided a summary and a comparison of existing similar work and summarized the recent research by highlighting the main contributions and application areas.
• We examined several important challenges and how to overcome such challenges, recent developments, and future opportunities in the studied research work; so that our readers and future researchers may better comprehend the present state of academic accomplishments on this subject.

### 1.2 Outline of study

To provide a comprehensive overview of our main subject: smart agriculture, the remainder of the paper is organized as follows. Following the introduction, section two provides a comprehensive overview of IoT in smart agriculture, highlighting its architectural elements, technologies, and the resources it was made out of. Afterward, section three summarizes the key application areas with their current status and recent developments witnessed. After section three, the latest advanced agricultural practices are highlighted in section four. Success stories of some countries that now excel well in smart agriculture have been shared in section five and the latest software and hardware utilized currently are highlighted in section six. Related research work and their contributions are highlighted in section seven. Section eight highlights the present status of smart agriculture and challenges concerning smart agriculture and how to overcome such challenges. Section nine highlights the anticipated future directions pertaining to smart agriculture and afterward, we conclude our research work with the conclusions we derived through our work. Table 1 showcase the acronyms used in the study.

**Table 1:** Acronyms found in this paper.

Acronym	Description
IoT	Internet of Things
AI	Artificial intelligence
GPS	Global Positioning System
M2M	Machine to Machine
CAPEX	Capital expenditures
OPEX	Operating expenses
SCB	Single-board computers
WSN	Wireless Sensor Networks
GPRS	General packet radio service
2G/3G/4G/5G/6G	Second-generation communication/Third-generation communication/Fourth-generation communication/Fifth-generation communication/Sixth-generation communication
LPWAN	Low Power Wide Area Networks
MQTT	Message Queuing Telemetry Transport
CoAP	Constrained Application Protocol
TCP	Transmission control protocol
IP	Internet protocol
UAV	Unmanned aerial vehicle
WSN	Wireless Sensor Network
FAN	Farm Area Network
GDP	Gross Domestic Production
NDVI	Normalized difference vegetation index
DDOS	Distributed Denial of Service
ML	Machine learning
DL	Deep learning
NFV	Network Function Virtualization
SDN	Software-Defined Networking

## 2 IoT in smart agriculture

The term “Internet of Things” most popularly known as IoT, was first used by Kevin Ashton, director of the Auto-ID Lab at the Massachusetts Institute of Technology (MIT), in 1999 [9]. Many researchers have defined IoT using different terms; references [6,7,9] refer to IoT as a series of interconnected devices that can intercommunicate with each other, while reference [10] describes IoT as the technology and, an environment in which sensors are placed at various objects, with the capability to share data in real-time via Internet connectivity. In simplest terms, IoT is a network of devices that use wired and wireless Internet to communicate with each other using Machine to Machine (M2M) communication, forming a ubiquitous network. Any device that has the capability to establish a connection with the Internet can be considered an IoT device; for instance, household appliances, surveillance cameras, furniture, and even people [[8], [9], [10], [11], [12], [13]]. To send and receive data, devices linked to the Internet generally require human intervention. This is in contrast to an IoT device which uses various networking protocols to enable the interchange of information between objects without the need for human intervention, offering ubiquitous connectivity and smooth operation. Thus, IoT technology has been used in a variety of application domains such as healthcare, cities, surveillance, military, transportation, manufacturing, and so on; due to these benefits [1,9,[11], [12], [13],[31], [32], [33], [34], [35]].

At a critical juncture in which most agricultural operations are hampered by factors such as global pandemics, higher capital expenditures (CAPEX) and operating expenses (OPEX), reluctance to use novel technology, scarcity of trained people, and low perception of people on agriculture-related works as a source of livelihood, IoT as a novel, innovative technology offers convenient solutions to all of these underlying problems through smart agriculture, revolutionizing the traditional form of agribusiness. IoT uses a variety of smart sensing devices to coordinate the majority of fieldwork and includes a wide range of components with features such as portability, high mobility, high precision, and a fast response that allows for faster processing, monitoring, and control capabilities, in order to mitigate the challenges associated with crop growth and subsequently, has a higher crop yield [6,7,36,37].

**Fig. 3:**
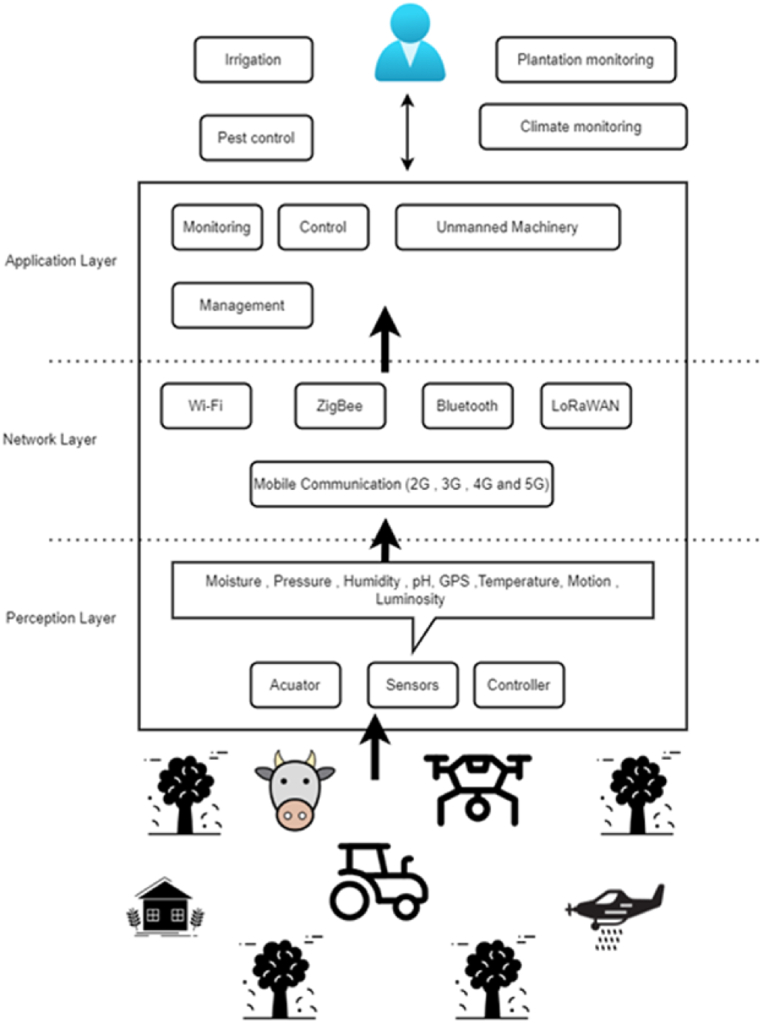
IoT solution architecture in Smart Agriculture.

**Fig. 4:**
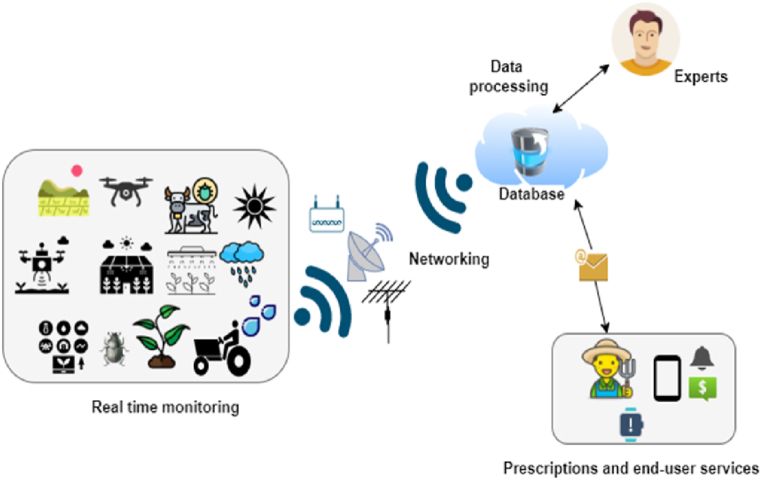
Procedures in smart agriculture.

Many researchers have understood that IoT architecture is much similar to the architecture of other computing systems, but the fact is that IoT has its own attributes that differentiate it from other computing systems, such as limited computing capability and its ubiquitous nature. The IoT architecture can generally be divided into three layers [[3], [4], [5],[7], [8], [9]], as shown in Fig. 3; although some researchers [6,10] have discussed a four-layer architecture, with a presence of an intermediate service layer that can further filter out and process the data gathered from IoT sensing devices. In a three-layer architecture of a typical IoT solution, the physical layer holds the responsibility for connectivity of physical devices and recognition; the network layer for data reception and transmission; the application layer for data offloading and offering various applications which end-users can interact with. In general, IoT sensor nodes can be put in various regions in the farming field, for instance, farming land, crops, irrigation systems, livestock, unmanned machinery, and greenhouses, to detect diverse parameters in real-time, and actuators control these parameters according to decisions taken at the application layer. For instance, smart agricultural solutions developed with IoT sensors and single-board computers (SCB) such as NodeMCU, Arduino, and Raspberry Pi can monitor various environment variables and can offer insights on soil moisture level, plant diseases, crop condition, soil condition, and soil pH level [3,[8], [9], [10], [11],[20], [21], [22], [23]]. The measured data are then sent to the local gateway, which are then uploaded to the cloud or remote servers, utilizing Wireless Sensor Networks (WSNs) at the network layer with the aid of various networking and application protocols [3,10,15]. This system may be used in a variety of agricultural applications, such as monitoring, control, unmanned machinery, and overall management of farms [3,6,[9], [10], [11]]. For better understanding, Fig. 4 depicts the procedures in smart agriculture where real-time gathered data are sent to the cloud or for expert opinion through the local gateways in the network layer. In the upcoming sections, these different layers are discussed further in detail.

### 2.1 Perception layer

The main function of the perception layer is to recognize the physical parameters of the target (e.g., farming land, livestock, crops, etc.) and provide connectivity to the sensing devices [3,10]. Various environmental variables have to be considered, such as air, soil, water, indoor (e.g., greenhouse) and outdoor (e.g., farming field) environment, atmosphere, etc., and various actuators are used to control these variables. The main sensors used in the context of smart agriculture include temperature, pH, humidity, moisture level, distance, light insensitivity, and motion sensors [3,[8], [9], [10], [11],[20], [21], [22], [23]]. Table 2 categorized the sensors used in the perception layer [[3], [4], [5],^9^,^12^].

**Table 2:** Sensors used in the perception layer.

Type of sensors	Main function	Application
Photoelectric sensors	Analyze signal by photoelectric effect	Monitor temperature, illumination, humidity
Location sensors	Locate precision location	Locate the precise location of crops
Mechanical sensors	Convert the measured physical quantity to mechanical quantity	Tensiometers to detect the force used by the roots in water absorption
Electrochemical sensors	Detect specific ions in the soil with electrodes	Monitor humidity, temperature
Airflow sensors	Convert a gas volume fraction into a corresponding electrical signal	Measure compaction, structure
Bio sensors	Exploit features of nanomaterials for different purposes	Analyze water, soil nutrients, soil humidity, pesticides, plant pathogens

### 2.2 Network layer

**Fig. 5:**
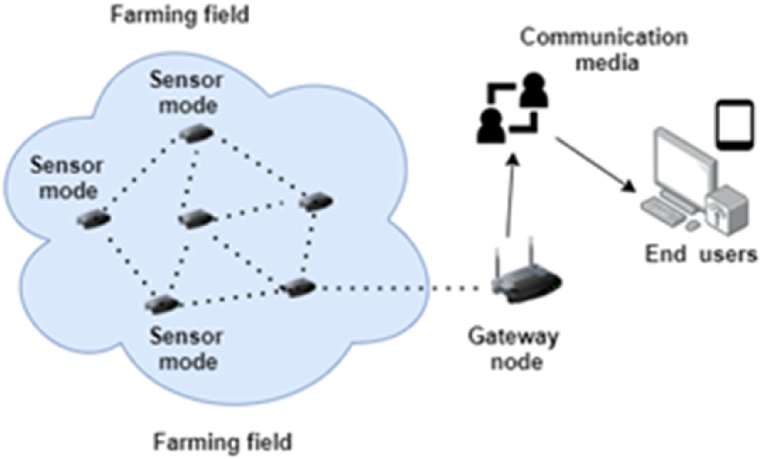
Wireless sensor network in smart agriculture.

The network layer holds the responsibility for processing and sending out the real-time data received from the perception layer to the application layer through wired and wireless networking media with the aid of different networking and application protocols [3,6,7,12]. In this regard, different networking and application protocols are used to facilitate communication, guaranteeing smooth connectivity and seamless operation [4,6]. These protocols are further used to establish WSNs, as shown in Fig. 5, which allow sensor nodes and applications to communicate wirelessly [[37], [38], [39], [40]]. Naturally, these varying protocols have different characteristics, such as power consumption, data transfer rate, and working range [[30], [31], [32], [33]]. Based on their characteristics, these networking protocols in the networking layer can further be apportioned into three types: cellular networks, short-range, and long-range [10,12].

Cellular networking protocols (e.g., GPRS, 2G, 3G, 4G, 5G, and 6G) offer communication over long distances and a higher data transmission rate than other networking protocols. On the contrary, the power consumption is too high for cellular networking protocols [[3], [4], [5], [6]]. Short-range networking protocols (e.g., Bluetooth, Wi-Fi, ZigBee) enable communication between shorter distances only and are used for communication between devices that are located close by Refs. [[3], [4], [5], [6],^12^]. However, they offer a higher data transmission rate despite low power consumption. On the other hand, the long-range network protocols (e.g., LoRAWAN) enable communication over long distances. These protocols are used to establish Low Power Wide Area Networks (LPWAN) as the power consumption is relatively low. Even though they offer long rage communication facilities, the data transmission rate of these protocols is low and used only when there is a need to transmit a low amount of data over long distances [12,[19], [20], [21]]. Further attributes of the networking technologies are presented in following Table 3 for better understanding and comparison.

**Table 3:** Comparison of networking protocols used in IoT.

Attribute	Bluetooth	ZigBee	Wi-Fi	LoRa	WiMAX	Mobile Communication
**Standard**	802.15.1	802.15.4	802.15.4 g	IEEE 802.11 a, b, g, n	IEEE 802.16	2G (GSM)/2.5G (GPRS)/3G (UMTS, CDMA2000)/4G (LTE)
**Topology**	Star	Mesh, Start,Tree	Star	Star	Start, Mesh	–
**Cost**	Low	Low	Low	Low	High	Medium
**Frequency**	2.4 GHz	868/915 MHz, 2.4 GHz	2.4 GHz	133/868/915 MHz	2-66 GHZ	2.4 GHZ
**Data transmission rate**	1–24 Mbps	20–250 kbps	2–54 Mbps	0.3–50 kbps	1 Mbps-1 Gbps	2G: 50–100 kbps/3G: 200 kbps/4G:0.1-1Gbps
**Power consumption**	Medium	Low	High	Very Low	Medium	High
**Range**	8–10 m	10–20 m	10–100 m	Less than 500m	<50 Km	Entire cellular area

According to the above comparison presented in Tables 3 and it is evident that power consumption is comparatively high when the data transmission rate increases. LoRa has a low power consumption even though it has higher coverage and low data transmission rate, which would be the most suitable for deploying in outdoor environments. For smart agriculture, these characteristics are highly useful when there is no proper energy supply in the field and when there are a lot of hindrances to wireless communication, such as adverse natural conditions and electromagnetic fields in the outside environment. Star, Mesh, and Tree topologies are used to implement these networks depending on the context [3,6,10]. In star topology, there is one central node and several peripheral nodes, with the peripheral nodes sending data directly to the central node for routing. On the other hand, a tree topology comprises leaf nodes and router nodes where each branch of the router node can be considered as a single star topology network [6]. In a mesh topology, each node can act as a router with rerouting capabilities, where messages in the mesh network are routed hop by hop until they reach their destination [6].

### 2.3 Application layer

The application layer holds the responsibility for archival, storage, process, analysis, and presentation of data that are received from the networking layer [3,9,[36], [37], [38],[40], [41], [42], [43], [44]]. In the application layer, application protocols such as Message Queuing Telemetry Transport (MQTT) and Constrained Application Protocol (CoAP) are used to send the data from source to destination between the end devices and the network layer. MQTT is a lightweight open-source messaging protocol that allows communication between IoT devices on unstable networks. It operates via TPC/IP or similar protocols, making it suitable for various IoT solutions [[8], [9], [10], [11], [12]]. It works like a “***Bulletin board***” where one device posts a message, and others can subscribe to receive it. This is particularly useful in agriculture for systems where sensors and devices (eg: soil moisture sensors or weather stations) need to send data to a central system or to other devices, even in remote areas with weak connectivity. MQTT helps farmers monitor and manage their farms more efficiently by enabling real-time updates and alerts for things like irrigation needs or the status of the underlying environment, even when network conditions are poor.

CoAP, like MQTT, is another communication protocol used for low-power devices that need to exchange data, especially in situations with unreliable networks. It operates like a ***“Telephone call***” where devices must first establish a connection before sending data. CoAP is great for small, energy-efficient devices like remote sensors in agriculture, such as those monitoring crop conditions or livestock health. These devices often rely on CoAP to send essential data back to a central system for analysis, which helps farmers make quick decisions about irrigation, pest control, and other vital farming activities [3,6]. Because the connection is “one-to-one” according to the client-server architecture, the usage of CoAP produces a strong coupling between the device that transmits messages and the device that is anticipated to receive messages [[8], [9], [10], [11], [12]].

In general, the application layer provides various intelligence services that end-users can interact with and help them to make precise decisions at the correct time. In this regard, big data allows distributed storage and parallel data processing, enabling the extraction of information in the shortest possible time, and such models are then used by AI models powered by Machine Learning (ML) and Deep Learning (DL) to identify the patterns, find the correlation, cluster the data and for forecasting and suggest prescriptions for end-users, using both structured and structured data [5,7,12,44,47]. Table 4 briefly compares major cloud IoT platforms that offer convenient services for processing the data at the application layer, in the context of smart agriculture.

**Table 4:** Comparison of growing cloud IoT platforms.

Cloud IoT platforms	Web Link	Data Visualization	Real time data capture	Data analytics	Cost per use
Thing Speak	https://thingspeak.com/	✓	✓	✓	free
Ubodots	http://ubidots.com/	✓	✓	✓	free
Nimbits	www.nimbits.com/	✓	✓	✓	free
Xively	https://xively.com/	✓	✓	x	free
ThingWorx	https://developer.thingworx.com/en	✓	✓	✓	Pay as you go
Phytech	http://www.phytech.com/	✓	✓	✓	Pay as you go
Yaler	https://yaler.net	✓	✓	✓	Pay as you go

## 3 Latest applications of smart agriculture

**Fig. 6:**
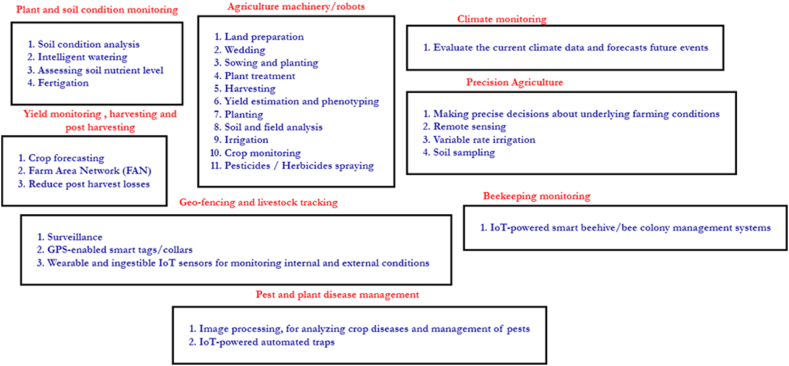
Summary of use cases of application we reviewed.

Recent advances in smart agriculture have paved the way toward overcoming many hindrances in traditional agriculture, making it super easy for farmers to manage their farms. Crop cultivation, on the other hand, is not as simple as scattering seeds and waiting for them to sprout; it is a time-consuming and resource-intensive undertaking that requires careful management [1,12]. The most recent technology developments in IoT-enabled smart agriculture include cheaper equipment, easier network connection, portability and durability, power consumption optimization, and hardware size reduction, making it a significant technological breakthrough in recent years [9,10]. In smart agriculture, IoT solutions and other enabling technologies are used to manage various aspects of agriculture farmland, from the preparation of land and seeding to sending harvest to the market [[47], [48], [49], [50]]. These IoT solutions are capable of offering a wide range of applications for improving crop yield and eliminating wastage. However, we have noted through the literature that a few researchers have attempted to categorize these different applications based on various attributes. In Ref. [9], the researchers have categorized applications into four categories: control systems, unmanned machinery, monitoring systems, and management systems, whilst in Ref. [10], the authors have categorized these application scenarios into outdoor and indoor environments. Outdoor agriculture includes environments such as orchards and arable land, whilst indoor agricultural environments include hydroponics, greenhouses, and crop beds and applications that serve in multiple environments. In the following, we outline the latest applications of smart agriculture in terms of their importance and popularity in recent years. Based on that we have categorized the uses of each individual application as depicted in Fig. 6, for providing a better overview.

### 3.1 Plant and soil condition monitoring

**Fig. 7:**
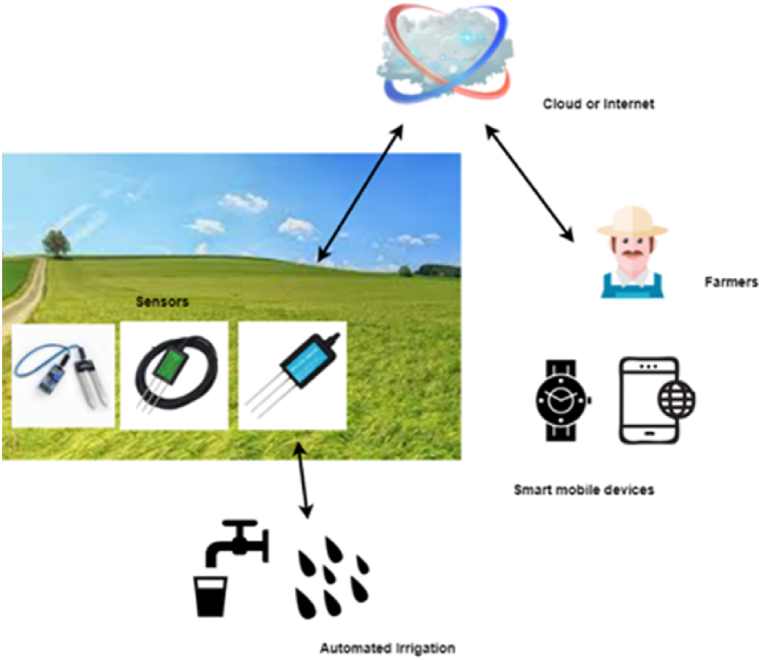
Soil condition monitoring and automated irrigation.

For a plant to grow and to have a better harvest, providing healthy soil is a must, as soil provides the necessary resources for a plant to grow by acting as an energy store that the plant needs [[1], [2], [3]]. In particular, soil anchors a plant's roots, supports its water supply, helps it breathe, and offers the nutrients it needs to grow in optimum conditions resulting in optimal plant growth in the expected duration [10,12]. Thus, to have optimum plant growth, which eventually leads to better harvest, farmers may place various sensors all around the field or embed them into the soil to analyze information about soil moisture condition, soil temperature, NPK (nitrogen, phosphorus, potassium) level, plant growth (e.g., the height of the plant), etc. [3,5,7,[9], [10], [11], [12]]. This has been made possible owing to the easy-to-use, cost-effective, and portable IoT sensing devices [12], which don't need extra Wi-Fi, or GSM, where it automatically reports the gathered data directly to the Internet [7], allowing farmers to monitor the condition in real time. Once the data is gathered, they are sent to the cloud for further analysis or expert opinion, where farmers can eventually get to know whether moisture, temperature, or growth level are optimal, low, or high through their smart mobile devices, which then can be controlled to optimum levels remotely as shown in Fig. 7.

Further, intelligent watering systems powered by IoT sensors can offer a certain level of automation, resulting in the conservation of water [[7], [8], [9], [10], [11]], and traditional irrigation methods are expected to change as a result of this utilization of IoT technologies. Conventional irrigation methods such as flood and furrow irrigation tend to waste more water as compared to novel controlled sprinkle and drip irrigation methods [6], which can be automated with the help of IoT. This is often done by changing the pre-specified watering schedule according to the underpinning recommending algorithm. When there is more soil water after rainfall, delayed or reduced amount of water can be supplied to the plants, and when the soil lacks soil water, water can be provided until the soil becomes concentrated with the required amount of water. In general, crop quality and quantity decrease when there is a water shortage, whilst excessive irrigation depletes soil nutrients and creates microbial infections [[9], [10], [11], [12], [13], [14], [15]]. As a result, precisely estimating crop water demand is challenging when factors such as crop type, irrigation strategy, soil type, precipitation, and soil moisture retention are considered. Given this, a precise soil and air moisture management system based on wireless sensors not only maximizes water efficiency but also improves crop health [6,9,12]. On the other hand, the most recent method of watering crops is called fertigation, and it involves injecting fertilizers, soil amendments, and other materials that are frequently required by farmers directly into the soil through the process of irrigation. Farmers are now able to regulate remotely how much fertilizer is injected and in what proportions thanks to fertigation systems that are enabled by the IoT [12].

Aside from soil and water, fertilizer is essential for plant growth. Natural or chemical substances combined with fertilizer provide crucial nutrients for plant growth. Plants primarily require three macronutrients: nitrogen (N) for leaf growth, phosphorus (P) for root, flower, and fruit development, and potassium (K) for stem growth and water movement [5,6]. Any nutrient deficiency or improper distribution can severely harm the plant's health [[5], [6], [7], [8], [9]]. More importantly, excessive fertilizer use, costs money and harms the land and ecology by eroding soil quality, contaminating groundwater, and contributing to global climate change [[19], [20], [21], [22], [23]]. Crops absorb less than half of the nitrogen fertilizer applied, with the remainder released into the atmosphere or lost through runoff. Fertilization in smart agriculture allows for precise nutrient requirements prediction, reducing their environmental impact [6,9]. Proper fertilization necessitates site-specific soil nutrient level assessments based on various factors such as crop type, soil type, soil absorption capacity, fertility type, utilization rate, and weather. This is due to the fact that measuring soil nutrient levels is both costly and time-consuming, as it frequently necessitates the analysis of soil samples at each location. Many IoT solution providers are now offering reasonably priced sensors that monitor soil humidity/moisture levels and soil/air temperature, which automatically reports back to farmers [33], keeping a constant eye on soil quality, assisting in water conservation, and reducing most operational costs in the long run [[26], [27], [28], [29]].

### 3.2 Agriculture machinery/robots

**Fig. 8:**
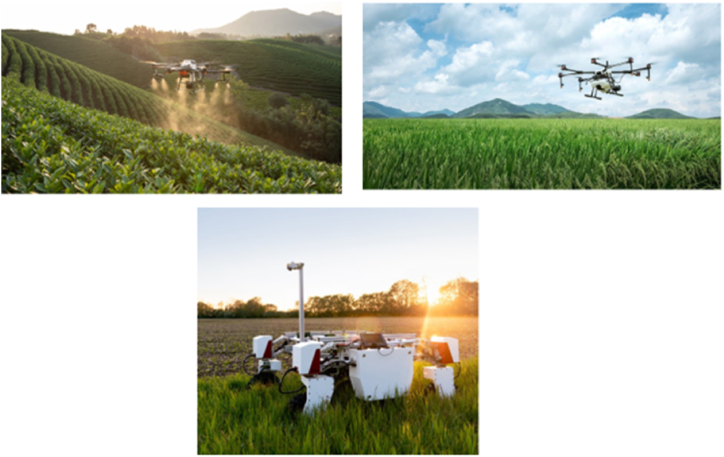
Agriculture robots in smart agriculture.

Agricultural robots are highly specialized technological devices that may help farmers with a range of activities. They can analyze, consider, and carry out a wide range of agricultural operations, and they can be programmed to grow and adapt to meet the needs of a variety of jobs [12,19,[21], [22], [23], [24], [25]]. The recent advancements in agricultural robots paved the way toward overcoming many challenges associated with rapid urbanization, shortage of skilled labour, adverse weather conditions, high competitiveness in the industry, and environmental pollution. The agriculture robots in smart agriculture can mainly be categorized into several categories based on the steps these machineries are involved in agriculture [12,19,21,37], as follows. Fig. 8 depicts some examples of how agriculture machinery can be used in the field.

• Agriculture machinery/robots for land preparation
• Agriculture machinery/robots for weeding
• Agriculture machinery/robots for sowing and planting
• Agriculture machinery/robots for plant treatment
• Agriculture machinery/robots for harvesting
• Agriculture machinery/robot for yield estimation and phenotyping

The important fact is that agricultural machinery has yet to be commercialized owing to the risk of accidents when the vehicle is unmanned. To deploy autonomous agricultural machinery in the field and ensure the safety of agricultural machinery and farmers, IoT technology and GPS should be combined [6,12]. Furthermore, most countries that use smart agriculture have a lot of croplands spread out over wider areas (e.g.: India, Australia, and New Zealand). As a result, utilizing self-driving autonomous machinery on these huge farmlands is a very cost-effective solution. In this regard, Unmanned Aerial Vehicles (UAVs), also referred to as drones, are used to enhance crop yield and monitor crop growth in these vast farming regions [21,37], where they have entirely revolutionized the way agriculture and farming are developed and done. Fertilizer sprinkling, crop monitoring, land monitoring, field research, and many more uses have all benefited from this drone technology [[3], [4], [5], [6],^12^,^21^,^37^]. In such scenarios where there are no communication facilities on the land and in vast agricultural areas, drones offer great benefits, as they can visit and communicate with wireless sensors placed over these farming regions [21,37]. Overall drones, equipped with high-resolution cameras and precise sensors, can fly over tens of thousands of hectares of farmland.

Obtaining aerial photographs of a field area now relies primarily on two methods: satellites and airplanes. Both are fine for a macro view of a landscape, but the quality of the photographs taken is becoming too bad when it comes to micro view [3,6,37]. Often, these macro-view photos are low-resolution and lack the image quality needed for further analysis, and decision-making, requiring frequent visits. Hence taking and collecting photographs on a regular basis is difficult with these conventional methods. Another serious concern is that both satellites and airplanes function above the cloud level, which means that in poor weather, their operations might be hindered, which ultimately paved the development and thriving of UAVs. With regard to agriculture robotics, Oliveira et al. [21] provided a comprehensive review of agriculture robotics and the key challenges ahead, pointing out that UAVs have the potential to leverage smart agriculture to the next level.

Further benefits of adopting UAVs in smart agriculture are described as follows for better understanding.

• Planting

Owing to human inaccessibility or a lack of appropriate personnel, millions of acres of land are now underutilized. The primary reason for not using these places for agriculture is safety concerns due to the rugged terrain. Drone-based planting systems are being developed for this purpose [[19], [20], [21]], which shoot pods with the seeds and nutrients needed to grow the plant, and this has been almost effective for rough terrain with an 80 % of success rate, according to the studies [19,21,37].

• Soil and field analysis

Drones can generate accurate information to examine the soil before seeding the crop, which aids in determining the best crop for a given plot of land, as well as recommending seed types and planting methods, according to the soil type [3,6,21].

• Irrigation

Equipping UAVs with a range of sensors and cameras can assist in identifying locations that are experiencing water stress and determining what irrigation adjustments are necessary. At the same time, they may be used to accurately spray water and pesticides on crops, which saves both time and waste, especially in emergencies [21,37].

• Crop monitoring

Crop monitoring is becoming challenging activity when the size of the farmland is large [[3], [4], [5]]. In this regard, drones regularly provide on-demand monitoring of remote farms, which is more cost-effective than satellite imaging [21].

• Pesticides/Herbicides spraying

UAVs can be used to spray herbicides and pesticides on crops in the same way as they can be used for irrigation [3,5,21]. Herbicides/pesticides are often sprayed throughout the farm, which is unnecessary for most circumstances as they may also damage crops. When spraying herbicide with a UAV, it may either spray directly on the undesirable weeds or target only the afflicted regions, which yields more precise results as opposed use of traditional ways of manual spraying [21,33,37]. Nonetheless, as drone spraying is extremely targeted and accurate, the drone would figure out and spray precisely as needed, lowering total consumption.

### 3.3 Climate monitoring

As the weather has a direct impact on determining the harvest, it is being used as an important parameter to evaluate the current climate data and forecasts future events beforehand so that farmers would be able to take necessary actions ahead, such as determining when to do the land preparation and harvesting [6,9]. On the other hand, when it's come to crop forecasting, which is a technique for forecasting harvest and assessing the quality and productivity before harvesting, it uses many agronomic and metrological data such as soil water holding capacity, to forecast an accurate state, which would always fluctuate with the climate conditions [12,[50], [51], [52], [53], [54]]. In smart agriculture, the local weather stations gather and process climate data from IoT sensing devices and deliver it to the farmers so that they can plan accordingly depending on the climate. Doing so will result in making the right decisions at the right time and help farmers lower their losses and improve the productivity of their harvest [[54], [55], [56], [57]]. In conclusion, climate monitoring allows farmers and other stakeholders to turn climatic threats into resources.

### 3.4 Precision agriculture

**Fig. 9:**
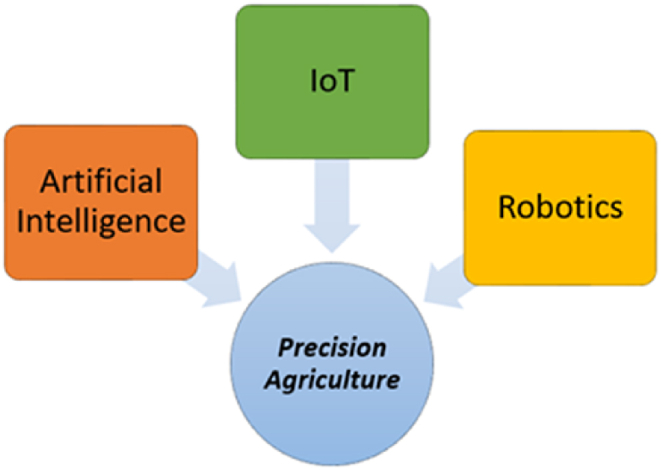
Composition of precision agriculture.

Precision agriculture, interchangeably known as precision farming, is the next big revolution of smart agriculture, where it has the capability to reshape the entire agriculture sector by aiding relevant stakeholders in making precise decisions at the right time [6]. According to Ref. [19], precision agriculture is defined as “A management method that gathers, processes, and analyses spatial and temporal data using electronic information and other technologies with the goal of guiding targeted actions that increase agricultural operations' efficiency, productivity, and sustainability”. From our point of view, precision agriculture can be known as agriculture that employs a variety of hardware, software, various control systems, sensors, autonomous systems, GPS systems, and other related technologies to offer precise and timely insights about crops and livestock, which eventually results in higher harvest, less wastage, and higher profit. Having said that, the key technologies in precision agriculture can be apportioned into three key categories, namely Robotics, IoT, and AI, as shown in Fig. 9 [[3], [4], [5], [6],^9^,^12^,^19^]. Most importantly, all these technologies can be used in tandem or separately.

According to the latest research [6,12,19], the market value of precision agriculture is estimated at approximately 8.5 billion in 2022, and it is estimated that it will reach more than 15 billion by 2030 [6,7]. In precision agriculture, IoT is used to gather data and agriculture robotics towards executing agriculture activities. In contrast, AI analyzes gathered data and makes precise decisions, making it the backbone of precision agriculture. In general, AI in precision agriculture is employed in broad disciplines of agriculture, offering analytical abilities in a wider area of smart agriculture, utilizing the data gathered from IoT sensors. Predicting the amount of harvest, climate condition for next month, what would be the best crop, profit, and loss analysis are a few application scenarios of precision agriculture [3,4,25,[55], [56], [57], [58]].

In this regard, the AI uses ML techniques, both supervised and unsupervised learning and DL, towards performing various classification, clustering, and correlation analysis tasks using both labeled and unlabelled data. Classification algorithms can classify and predict new occurrences, clustering is used to cluster data for identifying patterns, and correlation is used to determine the correlation between events. The classification algorithms include linear regression, support vector machine, and random forest, whilst clustering algorithms include k-nearest neighbour, k-means clustering, neural network, and so on [[25], [26], [27], [28],[30], [31], [32], [33], [34]]. Convolutional neural network, recurrent neural network, and generative adversarial network are a few examples of these DL algorithms.

On the other hand, robotics in precision agriculture is used to execute many of the agricultural tasks in the farming field, from land preparation up to the point of sending harvest to the market as aforementioned [19,21,37]. In straightforward terms, once the environmental data are fetched from IoT sensors located at the field, through the local network gateways, they are sent to the cloud or remote servers for further processing, where AI algorithms are used to make precise decisions and order agriculture robots to execute tasks based on the precise decisions taken [[58], [59], [60], [61], [62]]. The key applications of precision agriculture include remote sensing, variable rate irrigation, and soil sampling [12]. Remote sensing is the most effective approach to gathering information about an object without coming into direct contact with it, with the aid of WSNs; and could be utilized in smart agriculture to precisely obtain harvest information, such as the quantity that has been damaged and ramming, as well as to verify the status of the harvest. Sishodia et al. [25] provides an in-depth overview of remote sensing solutions in agriculture (e.g., solutions for irrigation, pest and plant disease management, yield prediction, crop monitoring, etc.), a variety of techniques, vegetation indices, and their latest applications. Variable-rate irrigation is used to preserve water in locations where water supplies are few and depends on topography, land level, and variable soil conditions [9,12]. Soil sampling is another precision farming breakthrough that can test the state of the soil to detect available nutrients in the soil, check the pH of the soil, and gather other important information linked to smart farming [12,37]. In their study, Monteleone et al. [55] have focused on adopting precision agriculture, focusing on farmer behaviour and operation management in the context of Agriculture 4.0 and Kayad et al. [61] provides an in-depth review of crucial precision agriculture applications in their study.

### 3.5 Geo fencing and livestock tracking

**Fig. 10:**
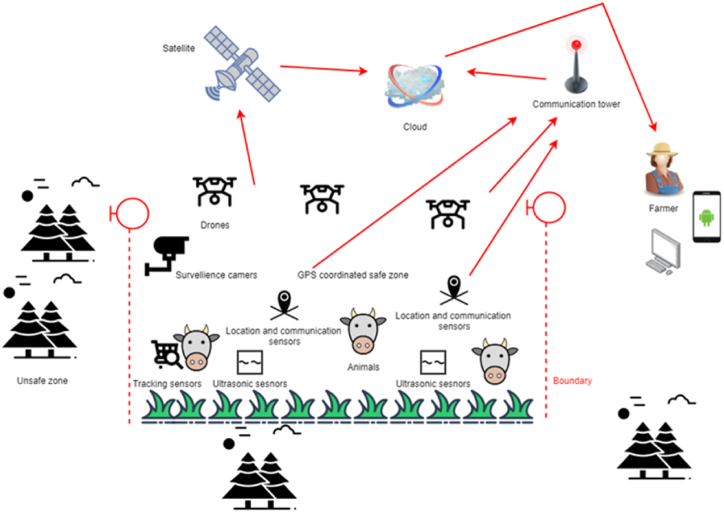
Livestock tracking and monitoring.

Domesticated animals reared in an agricultural context to generate commodities such as eggs, milk, meat, fur, and leather, are referred to as livestock [12]. The phrase is sometimes used to refer to animals kept for human consumption, while other times, it is only used to refer to farmed ruminants like cattle, sheep, and goats. Pork, veal, beef, and mutton are all classified as livestock by the United States Department of Agriculture, with all livestock classified as red meat [3,6,[12], [13], [14], [15]]. Due to the greater economic benefits of livestock in food production, protecting and monitoring livestock is essential for continuing these financial benefits. Farming fields and animals may be fitted with thermal imaging/night vision surveillance cameras, WSNs, monitoring devices, and tracking devices, allowing farmers to keep an eye on these gazing animals in terms of their security and health condition throughout their life span, as shown in Fig. 10. According to Ref. [12], smart tags/collars that use satellite technology (GPS) to geo-fence domesticated animals are now available, alerting farmers in real-time whether the animals in the herd have gone away or are at the boundary of a fenced zone, allowing farmers to know the real-time location at any time [12,63,67]. This will enable farmers to take a more educated and proactive approach to herd management, resulting in fewer animals being lost to predators or unfavorable occurrences [12]. These smart technologies might also track where the cattle go to eat and whether they are in a safe condition, helping farmers identify the most efficient use of grazing pasture and the safe deployment of animals. As of now, there are a variety of wearable and ingestible IoT sensors available to monitor the condition of livestock where internal sensors can be used to track digestion-related concerns, blood pressure, respiration rate, and other vital bio signs, while external sensors can be used to evaluate their overall health, detect injuries, verify breeding time, and analyze animal eating habits by watching movement patterns [12]. With all of the technology mentioned above, farmers can now monitor the status of their cattle in real-time and take precise interventions before things get worsen.

### 3.6 Beekeeping monitoring

**Fig. 11:**
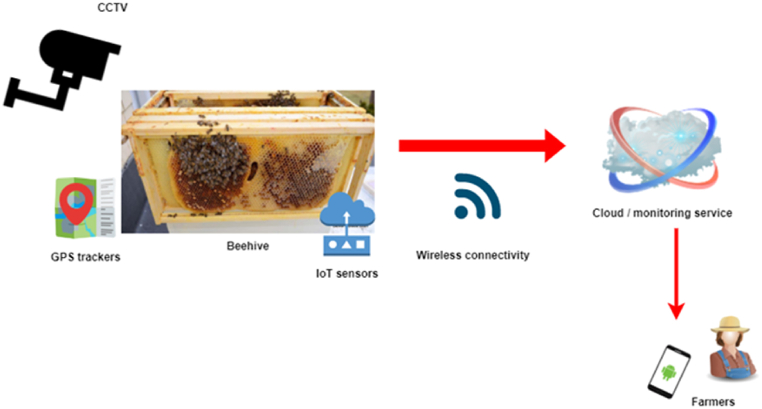
IoT for beehive monitoring.

With the increased demand for bee honey as a natural treatment and traditional herb, the global apiculture or beekeeping business is predicted to expand from $8 billion in 2019 to $11 billion by 2026. In beekeeping, bees are managed to extract honey and bee wax [38]. Bees not only offer wax and honey but also play a vital part in biodiversity preservation and maintenance of the natural ecosystem. They make honey and are important pollinators of fruit plants and crops, increasing agricultural yields and playing an important but undercover role in modern agriculture. Aside from parasites, pesticides, illnesses, and other stressors that influence bee health in this decade, beekeepers must also contend with the impact of climatic problems [39]. As a result, accurate and effective beekeeping monitoring systems are required for optimal bee colony management. With the advent of IoT-powered smart beehive management systems, beekeepers will be able to keep track of the quantity of honey produced in their hives and bee colonies even when they are far away from their hives through mobile phones, reducing the obstacles that beekeepers presently face. In general, nomadic beekeepers shift their hives according to the floral bloom to enhance honey output. Because of the distance of the apiaries, it may be more difficult for them to maintain control over the hives and respond quickly in the event of diseases or other stresses that influence bee health, whereas IoT-powered smart solutions may be more suitable for this [[68], [69], [70], [71], [72]]. Humidity, temperature, the system's overall weight, stored honey, disease outbreaks, and several other factors may all be utilized to remotely monitor and assess the hive's health with smart IoT solutions. In the case of theft or vandalism, the beehive is also fitted with a GPS tracker that can be used to trace it down. IoBee is a European Union-funded initiative that aims to disrupt the beekeeping industry by developing effective, timely, and user-friendly IoT monitoring technologies [40]. It focuses on the development and integration of several technologies to monitor pollinators and the environment they reside in. The sketch of an IoT beehive monitoring system is presented in Fig. 11.

### 3.7 Pest and plant disease management

Controlling the growth of crop diseases and pests is essential for farmers to have a better harvest, prevent the loss of crops, and maximize their revenue [33]. In general, pests can be defined as anything that people consider a threat to their lives, crops, animals, or other properties, such as viruses, bacteria, fungi, weeds, insects, etc. [3,6,7,12,19]. It is noted that, through the course of human history, several famines have happened caused by these pests. The Irish Potato Famine, which killed an estimated one million people in the 1950s, was caused by crop failure and production loss caused by the “potato blight” disease [[6], [7], [8], [9], [10]]. Moreover, it is estimated that 20 %–40 % of the harvest is lost annually due to pests and diseases worldwide [6,7]. Hence to control these pests and diseases and to prevent the loss of harvest, four methods can be used: chemical, cultural, biological, and integrated control [7], with the most effective methods in large-scale farming being chemical and integrated control as both biological and cultural methods require a lot of manual work. However, each method has its own pros and cons depending on the context [7,33].

**Fig. 12:**
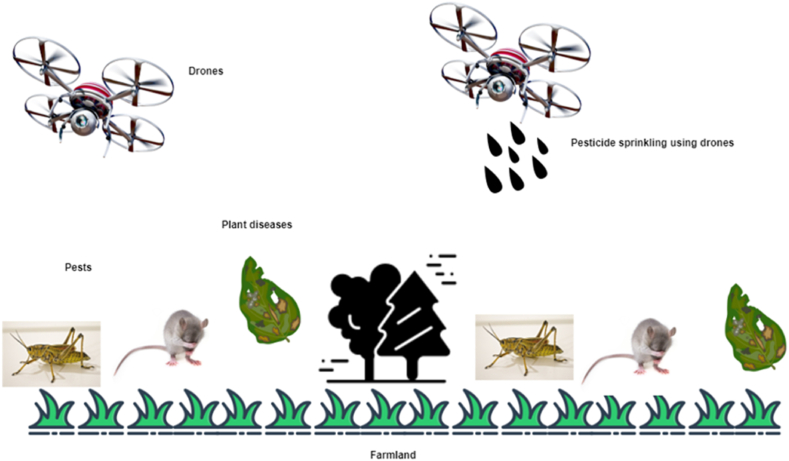
UAVs for real time pest and disease monitoring.

Since the beginning of the millennium, pesticides and other agrochemicals have become a significant part of the agriculture business. Each year, it is estimated that half a million tons of pesticides are used in the United States alone, with more than two million tons used globally [7]. The majority of these pesticides are detrimental to human and animal health, and they have a long-term, even irreversible, influence on the environment, contaminating the entire ecosystems. With recent advancements in agriculture robots, mainly the use of drones and WSNs, farmers can significantly reduce the usage of pesticides by accurately spotting crop enemies [6,7,[33], [34], [35]]. UAVs equipped with IoT sensors can monitor the incidence of pest and crop diseases from air macro and ground micro perspectives, as shown in Fig. 12 [[35], [36], [37]]. Modern smart agriculture provides effective pest and disease management procedure such as real-time monitoring, modeling, and forecasting future status and all these procedures depends on three aspects: sensing, evaluating, and treatment [7,36,37]. Image processing, in which raw images are acquired from the agricultural land for analyzing crop diseases and management of pests, using drones and remote sensing satellites is another advanced aspect of pest and disease control in smart agriculture [37].

Further, IoT-powered automated traps can capture, count, and recognize inspect types and can upload gathered data to the cloud for further analysis and notify farmers in real-time, in the event of any anomaly [6,8]. Under the control of remote IoT disease management systems, drones equipped with multispectral sensing devices and precision spraying nozzles may more accurately deal with pest problems by finding the correct target. Hence these IoT-based pest control systems offer many benefits, including the ability to lower total costs while also assisting in the restoration of nature back to its previous state [[34], [35], [36], [37],[72], [73], [74], [75]].

### 3.8 Yield monitoring and harvesting

**Fig. 13:**
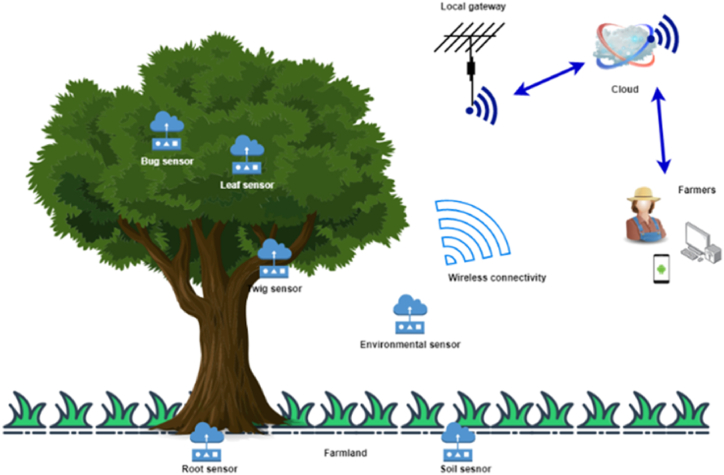
IoT-powered FAN.

Yield monitoring is an important aspect of modern agriculture as it can facilitate harvest improvement by considering many aspects related to yield, such as quality of the harvest and moisture content, towards having a competitive advantage in the market [3,5,6,55,[76], [77], [78]]. It assists in properly assessing crop yield and moisture level to determine how well the crops are and suggesting what to do next. Yield monitoring is considered an important aspect of smart farming both during and prior to harvest. On the other hand, crop forecasting is also an important aspect of smart agriculture which helps in predicting yield production (tons per hectare). It helps farmers in decision-making and planning for upcoming harvest [[7], [8], [9]]. Analyzing yield quality is another crucial aspect of farming that aid farmers in determining the right time for harvesting. In this regard, fruit size and color are taken as key inputs, and with these inputs, farmers can know whether the crops are ready to be harvested or not [[78], [79], [80], [81], [82]]. Further for continuous and real-time monitoring of the status of the entire farm, a Farm Area Network (FAN) can be set up and configured, covering the whole aspects of a farm [3,5,6], as shown in Fig. 13. In general, with FAN configured, it allows farmers to monitor the underlying conditions pertaining to the entire farm whenever they want.

In light of harvesting, there is a surge in demand for perishable foods (fruits and vegetables), especially in recent times owing to the quick pace of modern lifestyles. The rising demand results in an increase in profits; nevertheless, the management of food supply chains is faced with additional obstacles as a result of the vast amounts of food items and the greater diversity of food products. All these reasons can contribute to a major shortage of agricultural foods as well as a big retail loss, which is also referred to as post-harvest loss. This refers to the dramatic decrease in the quantity and quality of food produced between the time it is harvested and the time it is consumed, to put it in the simplest terms possible. The recent development of tracing and tracking technologies driven by IoT, AI, and blockchain, enable effective monitoring of the inventory and the quality of the products continuously until they are released to the market as end products. Not only that it also has the potential to significantly increase the efficiency of the food supply chain, minimize the amount of waste caused by perishable food products and tackle the challenges associated with food security [[78], [79], [80], [81], [82]].

## 4 Innovative smart agricultural practices

In the early days, farmers tried to enhance their harvest by focusing on using a variety of seeds, fertilizers, and pesticides [6,9,12,21]. However, these traditional ways have become insufficient for fulfilling the current demand, requiring innovative agricultural practices [[83], [84], [85], [86], [87]]. In order to tackle these issues, researchers have come up with alternatives such as bioengineered or genetically modified food [6], which is produced by modifying its DNA using genetic engineering. Even though their attempt has been helpful to some extent, several research studies have subsequently showcased the adverse consequences of this genetically modified food, including infertility, accelerated aging, and weakened immune system [[6], [7], [8], [9], [10], [11]]. Eventually, what happened was these technologies and their output did not receive much acceptance and appreciation from the global community, with people tending to prefer more natural and organic food over genetically modified food. Over the last few decades, a lot of research has been carried out to use IoT sensing technologies and other enabling technologies to transform the traditional way of farming to enhance the quality and quantity of the food while preserving its original nature in controlled environments, balancing the trade-off between external nature and the controlled environment. This has led to the development of novel agricultural practices such as greenhouse farming, vertical farming, hydroponic, aquaponic, and aeroponic [6,7,[40], [41], [42], [43], [44], [45],[87], [88], [89], [90], [91], [92]]. All these practices are now incorporated with smart IoT technologies for improving the quality and the quantity of harvest and have attained greater success. As of now, these technologies are used in many countries, particularly in countries with scarce resources, such as the United Arab Emirates, Taiwan, and Israel [[40], [41], [42], [43],[92], [93], [94], [95], [96]].

### 4.1 Greenhouse farming

**Fig. 14:**
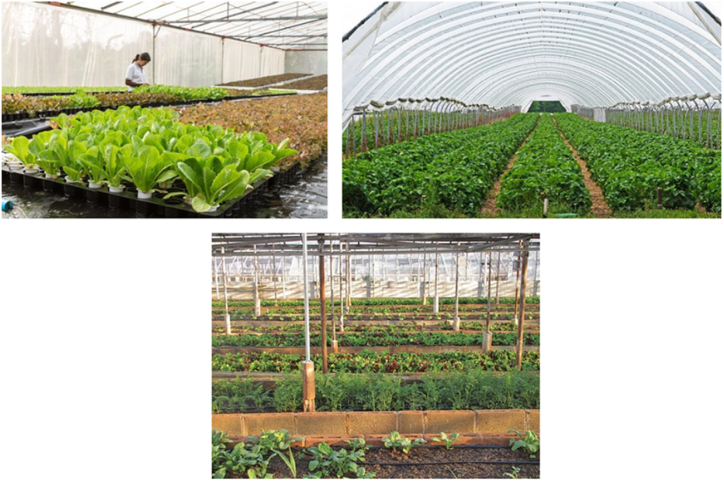
Greenhouse farming.

Carrying out farming activities inside a greenhouse is considered to be the oldest way of smart farming, where plants grow separately in an indoor environment without enduring the harsh and variable external environment, as shown in Fig. 14 [6,7,[96], [97], [98], [99], [100]]. The idea of growing plants inside an environmentally controlled area is dated back to the Roman civilization [41], and an actual greenhouse first emerged in England and Netherlands in the 17th century [41,42]. Having a long history back to the Roman empire, the greenhouse concept is becoming much more popular in countries with harsh weather conditions and scarce resources, such as in most Middle East countries. There are an estimated 7600 greenhouses in Abu Dhabi, the capital of the United Arab Emirates, which uses low to a high level of modern smarter technologies for its functioning [43,[100], [101], [102], [103]].

In greenhouse farming, crops produced inside are less impacted by the environment. They are not restricted to receiving light during the day, with artificial lighting conditions also provided during the nighttime [39,40]. This has resulted in higher harvest, and the crops which could previously be cultivated only in specific regions of the world under particular conditions now can be grown anywhere, anytime inside a greenhouse, under artificially controlled environments offering greater benefits for farmers as well as countries where the resources are scarce. In particular, when making these artificial conditions, IoT smart agricultural technologies are highly used for real-time monitoring and controlling the environmental conditions to an optimum level [[103], [104], [105], [106], [107], [108]].

### 4.2 Vertical farming

The decline of arable land has created an immense strain on meeting the current demand for food production as the amount of arable land is declining at a rapid phase owing to the rapid urbanization, erosion, and pollution [6,7]. Unfortunately, present agricultural practices are further degrading soil quality far quicker than the natural restoration processes, with erosion rates from cultivated fields estimated to be higher than soil formation rates according to the latest research [1,3,6]. On the other hand, with urbanization and environmental pollution, freshwater resources are declining rapidly, making it hard for farmers to carry out their farming activities [9]. These losses of arable land and limited access to freshwater resources have created immense pressure on meeting the growing demand for food production. Thus, vertical farming appears to be an innovative solution to these problems of land and water scarcity.

**Fig. 15:**
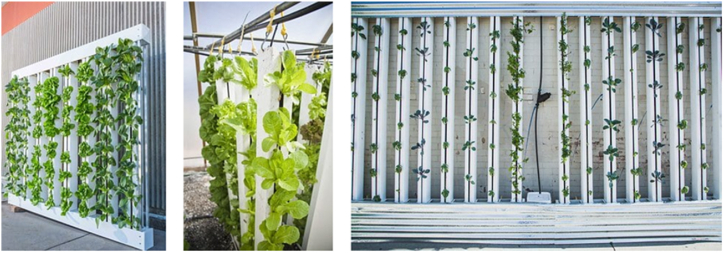
Vertical farming.

Vertical farming, as seen in Fig. 15, is a revolutionary form of urban farming in which crops are grown on top of each other (as stacked layers) in controlled conditions, resulting in reduced total resource use [[40], [41], [42], [43], [44]]. Vertical farming saves space while increasing crop yield per square foot of land, allowing farmers to improve food output. The method promotes soilless farming techniques, such as hydroponics, aquaponics, and aeroponics, which we will examine later in this section. Rooftops, tunnels, corridors, and buildings are some structures used to host these vertical farming systems. This makes vertical farming more suitable for urban environments, especially with the aid of smart IoT technologies, as more and more people in urban areas engage in vertical farming due to necessity despite the limitation on space [45,[102], [103], [104], [105],[109], [110], [111], [112], [113]].

### 4.3 Hydroponics

**Fig. 16:**
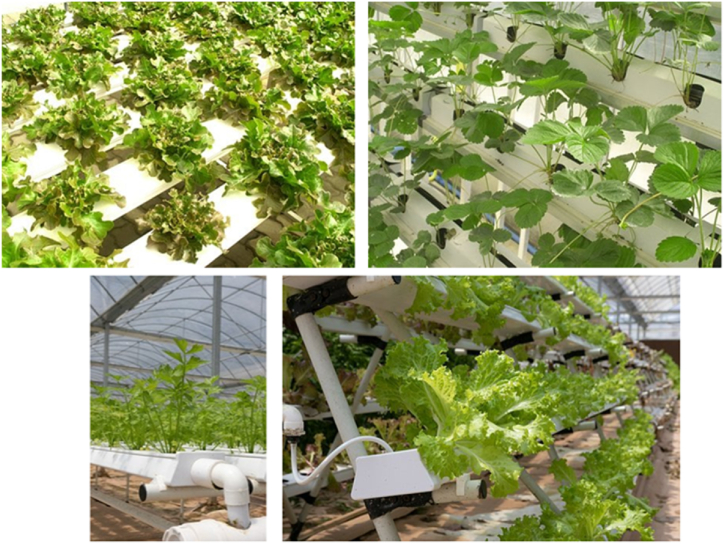
Hydroponics.

Hydroponic farming is an innovative way of farming where crops can be grown without the need for soil. It is considered a type of horticulture and a subset of hydroculture, where crops can be grown all year round as opposed to other traditional farming methods [3,46,[102], [103], [104], [105], [106],[113], [114], [115], [116]]. Traditional soil-based systems commonly consume more water than hydroponics farming when the plants are growing. On the other hand, hydroponic farming is based on a form of irrigation in which balanced nutrients are dissolved in water, and crop roots remain in that solution, with the roots supported by perlite or gravel [47]. Further, traditional soil-based plant growing techniques usually allow slower development of crops and less yield. In contrast, hydroponics farming solutions allow for higher growth of plants and more yield as the nutrients are directly dissolved in water, and roots can quickly grasp the required nutrients promptly [117,118]. Consumption of fewer resources, minimum space requirement, higher crop yield, and less maintenance are key advantages of hydroponics farming [[40], [41], [42], [43], [44]]. Fig. 16 depicts the typical setup of a hydroponic farming system. In the literature, Kout et al. [102] have proposed a hydroponic-based farming solution for saffron cultivation, one of the most expensive crops, using the NFT (nutrient film technique). Charumathi et al. [106] have proposed and evaluated the design of a hydroponic farming system that can allow farmers to grow their crops hydroponically.

### 4.4 Aquaponics

**Fig. 17:**
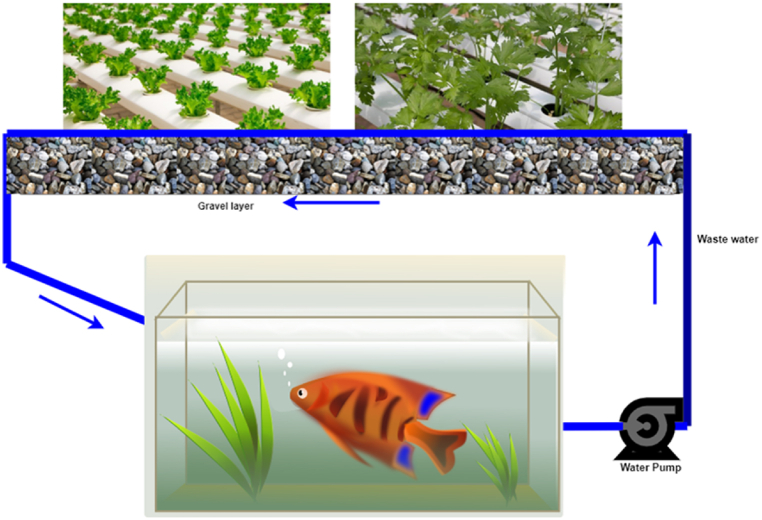
Aquaponics.

Aquaponics is a special form of hydroponic farming that combines aquaculture with hydroponic farming systems [[44], [45], [46], [47], [48]]. Normally aquaculture is referred to as raising aquatic animals such as fish, prawns, and snails in tanks for consumption or showcase [45,46]. Aquaponics combines hydroponic farming with aquaculture for growing plants in the water, with nutrient-rich water from a fish tank used to provide nutrition to hydroponically grown plants where nitrifying bacteria convert ammonia to nitrates [48,49], as shown in Fig. 17. This has been considered to be the best way of sustainable farming as nutrients are supplied through fish waste and has been used in both small and commercial food production purposes all around the world, with smart technologies employed to automate these farming systems [[102], [103], [104], [105],[112], [113], [114], [115]]. In this regard, Valiant et al. [113] proposed an IoT-powered automated aquaponics system using Nile Tilapia and Romaine Lettuce, capable of regulating temperature and pH level. Further, Tolentino et al. [115] presented a smart monitoring and automated system for an aquaponics set-up in a temperature-controlled greenhouse.

### 4.5 Aeroponics

**Fig. 18:**
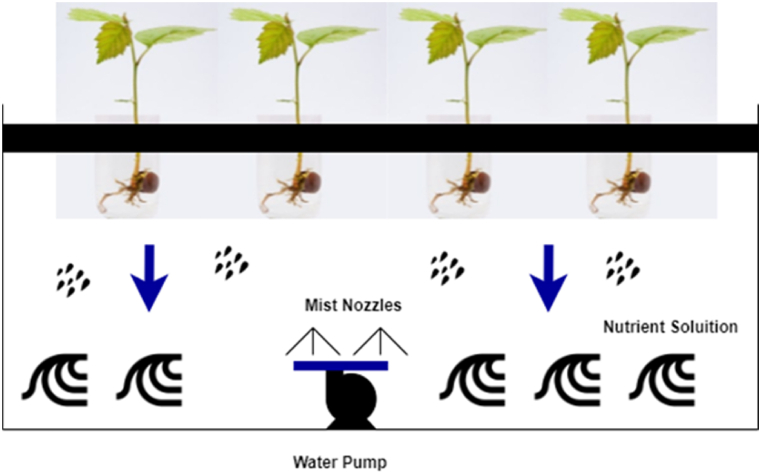
Aeroponics.

Aeroponic is another innovative farming method, where plants are grown without soil or aggregate media, often in mist environments or air, as shown in Fig. 18 [[46], [47], [48], [49], [50]]. The concept of aeroponic farming is based on hydroponic farming systems, where the roots of the plants are kept in soilless growth media, with nutrient-rich water pumped/sprayed over them regularly. This eliminates the need for a growth media similar to hydroponic farming. As shown in Fig. 18, aeroponic farming relies only on nutrient-rich mist to feed the roots of plants, which is provided through mist nozzles connected to the water pump [51]. As the nutrient-rich water is sprayed directly to plant roots, plants can grow to optimum maturity in the air with a plentiful supply of oxygen, water, and nutrients. Researchers such as Kerns & Lee [119] implemented a novel automatic aeroponics system using IoT devices, which provides a mobile application for users to monitor and adjust the aeroponic system. Jamhari et al. [120] designed a lab-scale IoT-powered aeroponic system capable of online monitoring and proved their system is ideal for vegetable plant growth. Further, Narimani et al. [121] developed an IoT-powered smart aeroponic greenhouse on an experimental scale and developed an AI-based system to control the environmental parameters inside the greenhouse.

### 4.6 Potential for innovative smart agricultural practices

While innovative smart agricultural practices such as vertical farming, hydroponics, aquaponics, and aeroponics have transformed farming by improving resource efficiency and yield, they are not without challenges. One of the primary concerns is the high initial cost of implementing these systems, including investments in IoT devices, sensors, and specialized infrastructure. For instance, setting up a hydroponic farm requires advanced equipment and expertise, which may be financially prohibitive for small-scale farmers.

On the other hand, another significant risk is the potential environmental impact of these technologies. For example, the increased reliance on energy-intensive systems, such as artificial lighting in vertical farming, can contribute to a higher carbon footprint if renewable energy sources are not used. Moreover, improper disposal of nutrient-rich wastewater from hydroponic and aquaponic systems may lead to water pollution [[112], [113], [114], [115]].

Additionally, there are technical and operational risks, such as system failures, dependency on consistent power and Internet connectivity, and the need for skilled labor to manage and maintain these technologies [[112], [113], [114], [115]]. Without adequate training and support, farmers may struggle to achieve the intended benefits of these innovations. Despite these challenges, continued research and development, alongside government and private sector support, can help mitigate these challenges making innovative agricultural practices more accessible and sustainable for farmers worldwide.

## 5 Success stories on smart agriculture

Having provided a holistic overview of smart agriculture, its latest applications, and innovative practices, this section is solely devoted to highlighting the use cases of smart agriculture as proof of evidence and providing guidance for future research. With this in mind, Israel and Taiwan have been selected as the two primary nations for which these use cases would be provided.

Israel is a very young and small nation, mostly covered by barren deserts; nonetheless, it has made more significant contributions to the field of smart agriculture than any other single country whilst achieving its dream of “Making the desert bloom.” Since 1950, they have found extraordinary ways to green their desert and spread their findings worldwide [122,123]. Growing food has always been challenging for Israelis because of the country's dry climate. It is estimated that around two-thirds of the country is classified as arid or semi-arid, and much of the soil quality is low. Precipitation is rare in the summer, and natural water supplies are severely limited [124,125]. Hence, Israel's agricultural industry has been on the cutting edge of innovation due to limited resources. As of now, the agricultural industry in Israel is currently second only to that of the United States [126]. Anyone who has had even a keen interest in contemporary agriculture is sure to have observed the rapid growth of smart agricultural advances in Israel. According to the World Economic Forum [[123], [124], [125], [126]], Israel is where businesses are most open to change and where most innovative enterprises are located because it invests 4.3 % of its GDP (Gross Domestic Production) on research & development, the highest percentage of any country. Not only that, but they also devote seventeen percent of the entire agricultural budget to research and development and the government works hard to foster strong relationships among the stakeholders in the agriculture sector [[123], [124], [125], [126]].

**Fig. 19:**
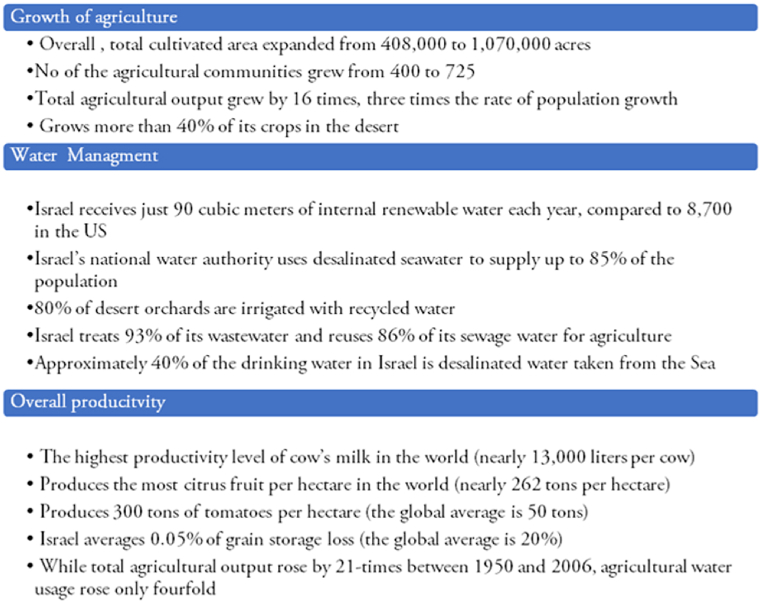
Latest statistics of growth and achievements of Israel's agriculture sector.

Growing crops in the desert is possible in Israel, owing to sustainable smart agriculture technologies that have transformed previously hostile regions into green breadbaskets. In addition, the government will maintain its efforts to find technological answers to climate challenges that heavily impact agriculture. To completely comprehend the modern-day miracle of Israeli agriculture, empowered by smart agricultural innovations, in Fig. 19, we highlight some useful insights in terms of how the country has overcome its problem of desertification, management of water supply and agricultural output, and achieved higher agricultural productivity through innovativeness and adoption of smarter technologies [[122], [123], [124], [125], [126]].

On the whole, Israel overcame the challenges of water scarcity, an inhospitable climate, and limited fertile land to become a world-leading agricultural powerhouse. Numerous Israeli companies produce tools for measuring, analysing, monitoring, and automating processes to give crops and soil exactly what they need and when and where they need it [[123], [124], [125], [126]]. This ensures minimal waste of resources while maximizing both efficiency and yield. One such disruptive Israeli innovation that changed the future of agriculture is the drip irrigation method, invented in Israel by Simcha Blass and his son Yeshayahu in 1959 to increase crop yield, quality, and consistency while using less water [[123], [124], [125]]. By far, this remains the foremost irrigation method worldwide, in the context of smart agriculture.

Moreover, Israel has developed innovative solutions, up to the level of watering crops with collected dew from the air and reducing the water needed for crops by almost 50 percent [124]. Based on their success, it is evident that three factors contributed a lot towards making Israel the world's leading agricultural powerhouse. First and foremost, the government's extensive support and commitment to research and development are making things easier, overcoming every obstacle on the way. Secondly, the country's volatile climate emphasizes the need for more self-sufficient farming methods and forces stakeholders to rely on smarter technologies like IoT and AI; and harsh natural conditions underscore the importance of ensuring enough supplies of both food and water. A third and final factor is the strong community support for adopting best practices and smarter technologies. Overall, the government connects all important actors in the agricultural community, including entrepreneurs, start-up companies, academics, investors, and service providers, to better understand their requirements and create operations around them.

Nonetheless, the government support, coupled with private sector innovations, has further driven its success. Companies like Netafim, the pioneer of drip irrigation technology, have transformed water usage in agriculture globally, helping farmers achieve higher yields with minimal water consumption [124]. Startups such as Taranis use AI and aerial imaging to provide early detection of crop diseases and pests, while CropX offers IoT-powered soil sensors that optimize irrigation and fertilization [[127], [128], [129], [130], [131]].

In addition to domestic innovations, Israel has actively engaged in international collaborations to expand the impact of its technologies. The Indo-Israel Agricultural Project (IIAP), for instance, has established Centers of Excellence across India, promoting advanced horticulture and water-efficient farming practices. Similarly, the USAID Middle East Regional Cooperation Program fosters research partnerships between Israel and neighboring countries, focusing on climate-resilient crops and sustainable resource management. These efforts highlight Israel's dual approach of fostering innovation locally and sharing it globally [[127], [128], [129], [130], [131]].

When it comes to Taiwan, an island nation located in East Asia, recent years have seen it achieve immense growth in the field of smart agriculture. It is estimated that Taiwan's agricultural, forestry, fisheries, and animal husbandry industries contributed around 18.6 billion USD to its GDP in 2021 [19]. Overall, the country possesses 1.9 million acres of arable land. Taiwan's economy is known primarily for its semiconductor industry and Information Technology. However, Taiwan's economic rise in the technological field was only possible because of a solid foundation in the agricultural sector [127]. Agriculture, which served as the backbone for growth in industry and commerce, became the starting point for Taiwan's economic development in the latter half of the twentieth century, and today, the agricultural sector benefits from the island's technological growth.

There is now a serious lack of labor on Taiwan's farms due to the country's low birth rate and rapidly aging population. On the other hand, problems caused by the volatile climate negatively impact agricultural productivity. Thus, the government of Taiwan has enacted laws to encourage “Smart Agriculture” to cope with the effects of climate change and make headway against the problem of a lack of available human resources. This encompasses programs that provide financial assistance through subsidies to farmers who are willing to adopt or create smart agricultural technology, goods, or services. Further, the government offers possibilities for farming enterprises that are competent and engaged in IoT, robots, drones, and AI, to extend their services and promote their solutions. Not only that, but the government has also developed various solutions to the problems that the agriculture business is currently facing, including remote process control, using IoT, and related enabling technologies. As a result, farmers can remotely monitor their fields using mobile devices and systematically connect their agricultural production with consumer markets [19,[127], [128], [129], [130]]. In addition, the government continues to use big data analysis to keep track of the conditions of its agricultural import and export markets [19]. This helps the government to direct the industry to make flexible adjustments to production in a timely manner and to keep production and sales stable at any given time.

**Fig. 20:**
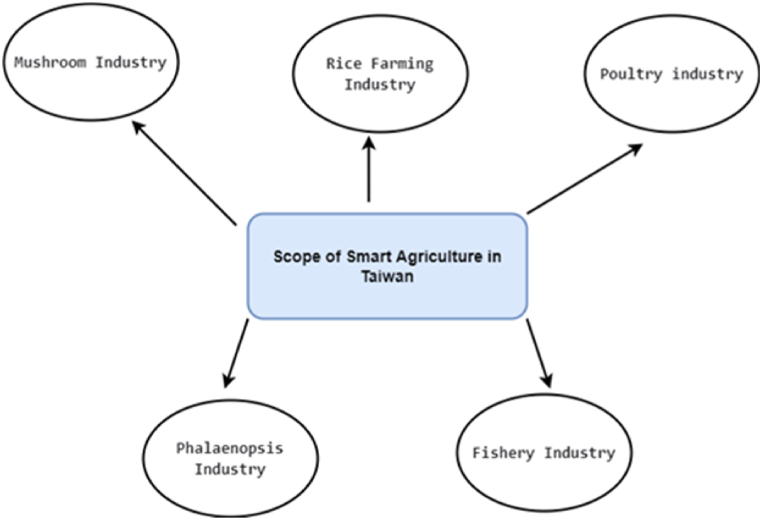
Prioritized industries by Taiwan towards promoting smart agriculture.

To promote smart agricultural practices, the Taiwan government has prioritized some of the agricultural industries [19,[127], [128], [129]], as shown in Fig. 20. With the government initiatives already started and planned for the future, the industries depicted in Fig. 20 would receive the highest amount of investment and resources to expand their capacity to the fullest potential.

Government initiatives state that habitats used for mushroom cultivation will be entirely automated, including regulating environmental factors, until the harvesting time. The creation of mushroom manufacturing facilities and associated machinery would allow for the integration of hardware and software and the introduction of energy-saving technology for mushroom cultivation, such as the use of solar energy. When it comes to rice farming, the government plans to establish an intelligent system and construct a human-machine collaboration and smart production of rice cultivation. With it, a new type of agricultural service business will be created, and the rice industry's efficiency and competitiveness will be increased, and producers and customers will be connected. Moreover, by developing a smart poultry breeding production management system and establishing a recognition and verification mechanism, the government plans to confirm; that poultry production conforms to international food safety production standards. In addition, the Taiwan government plans to export smart poultry breeding technology to assist other countries, so they can earn revenue through that. The intelligent technology used in poultry/poultry egg production can also be applied to other livestock sectors, which the government is currently planning as an initiative. When it comes to the phalaenopsis industry, which brings a lot of foreign revenue through exports, to eliminate the need for labor, the government emphasizes that the industry must adopt smart technologies for automated pot changing, watering, and spraying. With the adoption of IoT, big data, and related enabling technologies, the fisheries industry should build a dataset of fishery information and should, track the supply chain, regulate inventories and external sales on time, avoid production and sales imbalances, and maintain the industry's competitiveness through big data analysis as per the government initiatives [19,[127], [128], [129], [130]].

Use of artificial lighting to increase the harvest [19], through artificial control of environmental conditions (humidity, temperature, and airflow), mimic natural microclimates to provide optimal conditions for crops [128], use of IoT-powered safe poultry houses to automated dispense of food and water, retrieval of average weight gain of birds in poultry houses via IoT powered underfloor scales and establishing the most advanced digital farm in Asia with Artificial Intelligence of Things Technology (AIoT) [[127], [128], [129], [130]]; are some of the unique examples that showcase how Taiwan is excelled at these smart agricultural solutions.

Nonetheless, Taiwan's International Cooperation and Development Fund (ICDF) has been instrumental in promoting smart agriculture internationally, collaborating with developing countries in Asia and Africa. Through these partnerships, Taiwan has transferred technologies for automated irrigation, pest management, and precision farming [[127], [128], [129], [130]]. Additionally, the Smart Agriculture Technology Exchange Program with Japan has facilitated the exchange of innovations like AI-powered crop monitoring systems, benefiting both nations and reinforcing Taiwan's position as a leader in smart farming [[127], [128], [129], [130]].

Currently, hundreds of Taiwanese companies deliver intelligent farming components to the world, from semiconductor chips to microcontrollers and UAVs. The small island could become one of the world's largest and most profitable food producers within the next decade, owing to its technological infrastructure, the success of smart agricultural initiatives, its strategic location, and well-developed transport links.

## 6 Smart agriculture software and hardware

In this section, we highlight the latest versions of IoT software and hardware, which play a crucial role in strengthening the smart agriculture ecosystem by offering convenient solutions for all stakeholders involved in smart agriculture.

### 6.1 Smart agriculture software

To help farmers save time and money, the IoT integrates remote sensors, robots, and unmanned machinery with AI and computer software to monitor crop growth, conduct field surveys, and deliver data-driven insights to the farmers/farm management. Table 5 highlights the most well-known IoT software currently utilized in agriculture, considering both mobile and application software's together.

**Table 5:** IoT software's used in agriculture.

Software	Description
CropX	CropX is a cloud-based software that facilities ranchers to boost yield by focusing on saving water and energy. With the sensors placed on the field, farmers have full control over irrigation through a GPS-enabled mobile app [131,132].
Sirrus	Sirrus provides a mobile and web-based platform for enabling smooth collaborations between experts and farmers/growers by rendering the data from the agricultural field, facilitating both parties to share ideas/recommendations, and providing easy access to all stakeholders [131,132].
Cropio	Cropio provides Cropwise Operations, a decision-making tool that aims to optimize fertilization and irrigation, reducing the amount of fertilizer and water used. It combines weather and satellite data and allows crop monitoring and yield forecasting [133].
AgroPal	Agro Pal is an AI-enabled Android app that assists farmers with crop details, plant disease detection, and weather forecasting, as well as providing useful insights to farmers through community-powered forum discussions [134].
AgriXP	AgriXP is a platform for farmers and crop consultants to track field data. It enables farmers, employees, and crop consultants to record and export data from all agricultural field activities [135].
Nutrient ROI calculator	The nutrient ROI calculator by eKonomics is the first of its kind to cover major crops in most of North America. The most up-to-date ROI calculator accounts for spatial variation when calculating economic return, which aids farmers in optimizing yields and profits [136].
Farmbot	FarmBot is a precision farming solution built with open-source hardware and software. With the help of a robot, anyone can manage their garden from anywhere at any time using the FarmBot platform [137].
FARMapper	Farmapper is a cloud-based software platform for farmers and ranchers that allows farmers, ranchers, and landlords to create, build, and customize farm maps and share them [136].
AgVault 2.0	Drone collected, high-resolution, and NDVI (normalized difference vegetation index) data can be used by farmers on an unlimited number of acres with the help of AgVault software. Further, it allows you to navigate the UAV and control its position along with route planning [136].
Climate App	Using this app, farmers can keep a close watch on critical temperatures. Maps of current highs, lows, and ground temperatures are available in the Climate App. The goal is to inform farmers and agricultural workers about the temperature ranges that could affect their production schedules [138].
AGRIVI 360 Farm Insights	An intuitive farm management program that helps farmers make informed agronomic decisions based on current data from the field while also streamlining administrative tasks. Further software offers the facility for record-keeping, gathering real-time field insights, and allowing crop traceability [139].
FarmLogs	FarmLogs allows ranchers to collect and log information on a field-by-field basis. In addition, it facilitates budgeting and planning for each season and keeping tabs on seasonal lists and precipitation records [140].
Farm At Hand	Using Farm At Hand, a free, web-based farm management app, you can streamline your entire agricultural operation more easily and efficiently. The app also records crucial information from the field and lets you keep tabs on things like harvesting, spraying, and planting [136].
Farmeron	Farmeron is a platform that facilitates digitizing your dairy farm's records and improving those records through the use of actionable metrics and advice [141].
AgriSync	AgriSync facilitates farmers to connect with their advisors/consultants using the app instantly and makes a collaborative platform where farmers can share and sort out their questions via expert ideas [142].

### 6.2 Smart agriculture hardware

The most important component of a smart agricultural solution is its physical hardware, which is solely responsible for gathering data and keeping the solution operational. This section highlights some of the well-known IoT hardware used in agriculture by researchers and industry to develop smart agricultural solutions. In this regard Table 6 describes some of these commonly used IoT hardware.

**Table 6:** IoT hardware used in agriculture.

Hardware	Description
Raspberry Pi	Raspberry Pi is a small single-board computer (SBC) that is well suited for developing IoT owing to its small size and capabilities such as high processing power, memory, and strogae compared to its size.
NodeMCU	NodeMcu is an open-source firmware and development kit that facilitates to prototype of IoT. The device has built-in Wi-Fi capability and is much cheaper than other microcontrollers that can prototype IoT.
Ardunio Uno/Mega/Nano	Arduino Uno is a microcontroller that has 14 digital input/output pins. Arduino Mega is a microcontroller with 54 digital input/output pins, unlike Uno. Arduino Nano is a small breadboard-friendly board with fewer pins than Uno and Mega.
DHT11	The DHT11 is commonly used to measure temperature and humidity, and the output value can be obtained as serial data. The sensor can measure temperature from 0 °C to 50 °C and humidity from 20 % to 90 % with an accuracy of ±1 °C and ±1 %.
RTC module	RTC module is often used to set up a real-time clock and keep the time and date up to date. It has a battery setup that keeps the module running in the absence of external power.
GSM module	GSM module allows microcontrollers to connect to the Internet, send and receive SMS, and make voice calls using the GSM library.
Soil moisture sensor	Soil moisture sensors measure or estimate how much water is in the soil. These sensors can be fixed or mobile, such as handheld probes. Stationary sensors are installed in the field at predetermined locations and depths, whereas portable soil moisture probes can measure soil moisture at multiple locations.
LM 35	LM35 is a 3-terminal sensor that measures surrounding temperatures ranging from −55 °C to 150 °C.
pH sensor	A pH sensor measures the water's acidity with a value between 0 and 14. In smart agriculture, these sensors are often used to measure the pH of hydroponics and aquaponics systems and help farmers to balance the pH level constantly.
EC sensor	The EC sensor measures the electrical conductivity in a solution and is often used to measure the electrical conductivity of hydroponic farming and aquaculture systems and is also used for water quality testing.
Turbidity Sensor	Turbidity sensors measure the amount of light scattered in water by suspended solids and are used in river and stream gaging wastewater, and effluent measurements.

## 7 Related work and contributions

Having discussed the history, the main foundation, the architecture and its main components, the latest applications and advanced practices along with software and hardware that are utilized, and the latest innovations that drive the smart agriculture domain with success stories of some countries, it is now evident that smart agriculture is becoming the next big revolution in the world, contributing to eradicating the world hunger. The fact that smart IoT technologies can be applied in different steps of the agricultural process, from sowing to harvesting and delivering the end products to the market, has proven to the world the importance of adopting the technology for every aspect of human life. This has proven to be very effective and important during this COVID-19 pandemic time. In particular, as we observed, the main aim of smart agriculture is to improve productivity while utilizing minimum resources in an optimum manner, balancing the trade-off with nature, and eliminating overall wastage.

In this section, we mainly highlight what exact contributions have been made by other researchers toward the state of the art. In Table 7, we highlight the contributions made by others towards the state of the art, by highlighting the title, scope of the study, and key findings along with our observations, which can be used to compare the current level of the status of the state of the art.

**Table 7:** Summary of contributions.

Reference	Title	Scope of the study	Contributions and critique
(Yang et al., 2021) [4],	A Survey on Smart Agriculture: Development Modes, Technologies, and Security and Privacy Challenges	Smart agriculture/security and privacy challenges pertained to smart agriculture	In this review, the authors have summarized several modes of smart agriculture (e.g., precision agriculture and facility agriculture) and derived key applications and technologies. Further, they have provided a comprehensive review of prevailing security challenges of smart agriculture and highlighted countermeasures for overcoming such challenges as the key contributions of the study.
(Ayaz et al., 2019) [6],	Internet-of-Things (IoT)-Based Smart Agriculture: Toward Making the Fields Talk	Smart agriculture	The researchers have provided a comprehensive overview of smart agriculture and the obstacles encountered when wireless sensors and IoT are integrated with traditional farming techniques. In this regard, they have categorized sensors based on the agricultural application and discussed how these technologies help farmers in having a better crop yield. Future trends and the latest challenges are also highlighted but they have not highlighted how such challenges can be overcome.
(Mekala and Viswanathan, 2017) [7],	A Survey: Smart agriculture IoT with cloud computing	Smart agriculture	The researchers have surveyed the applications of IoT in smart agriculture that uses cloud computing as the major backbone of such solutions. Apart from IoT and cloud integration, they have not focused on any aspects of smart agriculture.
(Kim et al., 2020) [9],	A Review of the Applications of the Internet of Things (IoT) for Agricultural Automation	Smart agriculture	The researchers have summarized and reviewed the use cases of IoT applications applied toward agricultural automation and presented the prospects and limitations of using IoT in the agricultural sector in Korea.
(Navarro et al., 2020) [10],	A Systematic Review of IoT Solutions for Smart Farming	Smart agriculture	The researchers have used PRISMA (Preferred Reporting Items for Systematic Reviews) methodology to review the existing literature on smart farming with IoT in a systematic manner. In their systematic review, they have highlighted key technologies, platforms, and devices, networking protocols and further evaluated the applicability of IoT in the agriculture sector. However, the review is purely focused on the integration of IoT with agriculture and its uses.
(Madushanki et al., 2019) [11],	Adoption of the Internet of Things (IoT) in Agriculture and Smart Farming towards Urban Greening: A Review	Smart agriculture	The researchers have examined the latest IoT applications and provided an overview of key technologies, sensors, and applications used in agriculture. For their review, they collected data from 60 peer-reviewed scientific publications from 2016 to 2018.
(Thilakarathne et al., 2021) [12],	Internet of Things in Smart Agriculture: Challenges, Opportunities and Future Directions	Smart agriculture	The researchers have summarized the latest research that has been done and highlighted the key components, latest applications, challenges, and future direction in terms of smart agriculture. Further, the authors already have highlighted how COVID-19 impacts the smart agriculture domain after,2020.
(Oliveira et al., 2021) [21],	Advances in Agriculture Robotics: A State-of-the-Art Review and Challenges Ahead	Agriculture robotics in smart agriculture	The researchers have reviewed the applications of agricultural robot systems that can be used in farming, from land preparation to sending harvests to the market. In their review, they analyzed respective computer vision algorithms, navigation systems, and sensors used in these robotic applications. Their result suggests that investment in agricultural robot systems would produce both long (harvesting and yield estimation) and short-term (plant and harvest monitoring) results.
(Ullo and Sinha, 2021) [22],	Advances in IoT and Smart Sensors for Remote Sensing and Agriculture Applications	Smart agriculture/remote sensing	The authors conduct a comprehensive evaluation of current research and studies in the field of IoT and sensors used in remote sensing applications as well as precision agriculture.
(Sishodia et al., 2020) [25],	Applications of Remote Sensing in Precision Agriculture: A Review	Precision agriculture/remote sensing	The researchers provide an in-depth overview of remote sensing systems, a variety of techniques, vegetation indices, and their latest applications used in agriculture.
(Sinwar et al., 2020) [27],	AI-Based Yield Prediction and Smart Irrigation	AI for smart agriculture	The authors have provided a comprehensive review of AI for smart irrigation and yield prediction applications in the context of smart agriculture. The study only focuses on these two application areas.
(Garg and Alam, 2020) [28],	Deep Learning and IoT for Agricultural Applications	Smart agriculture and integration of AI (especially DL)	The authors have provided a review of the usage of DL applications in smart agriculture and potential solutions that can be solved by applying these DL application-based solutions.
(Zhu et al., 2018) [29],	Deep learning for smart agriculture: Concepts, tools, applications, and opportunities	Smart agriculture and integration of AI (especially DL)	The research provided a concise summary of DL applications in smart agriculture and highlighted key concepts, their implementation, limitations.
(Ferrag et al., 2021) [30],	Deep Learning-Based Intrusion Detection for Distributed Denial of Service Attack in Agriculture 4.0	Security of smart agriculture	The researchers have proposed an intrusion detection system based on DL for detecting DDOS (Distributed Denial of Service) attacks that target smart agriculture infrastructure.
(Dutta et al., 2020) [36],	Smart farming: An opportunity for efficient monitoring and detection of pests and diseases	Detection of plant diseases and pests in smart agriculture	The authors have reviewed pest and disease detection in smart agriculture.
(Pandey and Vamanan, 2020) [53],	Deep Learning and Internet of things Integrated Farming during COVID-19 in India	Smart agriculture and integration of AI (especially DL)	The authors explore how DL can be used to overcome problems in traditional agriculture in the context of India, owing to the growing demand for smart agricultural solutions in India.
(Monteleone et al., 2020) [55],	Exploring the Adoption of Precision Agriculture for Irrigation in the Context of Agriculture 4.0: The Key Role of Internet of Things	Precision agriculture	In the context of Agriculture 4.0, the researchers have focused on analyzing factors that affect the adoption of precision agriculture, focusing on farmer behavior and operation management.
(Salam and Shah, 2019) [58],	Internet of Things in Smart Agriculture: Enabling Technologies	Smart agriculture	The latest innovations and an overview of smart agriculture are presented in this study. However, the study is more focused on highlighting the necessity of smart agriculture.
(Sivakumar et al., 2022) [59],	Internet of Things and Machine Learning Applications for Smart Precision Agriculture	Smart agriculture and integration of AI (especially ML)	The researchers have provided in-depth insights on ML applications in smart agriculture and discussed challenges and solutions along with their implementations.
(Kayad et al., 2020) [61],	Latest Advances in Sensor Applications in Agriculture	Precision agriculture/remote sensing	The authors have provided a review of sensor applications used in the context of precision agriculture, highlighting examples of use cases.
(Liakos et al., 2018) [62],	Machine Learning in Agriculture: A Review	Smart agriculture and integration of AI (especially ML)	The authors have provided a review of ML applications used in smart agriculture. The study was sectioned into reviewing applications in terms of crop management, livestock management, water management and soil management.
(Sott et al., 2021) [67],	Agriculture 4.0 and Smart Sensors. The Scientific Evolution of Digital Agriculture: Challenges and Opportunities	Smart agriculture	The authors have provided a systematic review (bibliometric analysis) on smart agriculture in terms of agriculture 4.0 using the publications indexed on the Web of Science database.
(Demestichas et al., 2020) [68],	Survey on Security Threats in Agricultural IoT and Smart Farming	Security and privacy on smart agriculture	The paper first presents an overview of the use of ICT solutions in agriculture and then provides greater insights into existing and potential threats and vulnerabilities in smart agriculture. Further, they have highlighted mitigation approaches toward protecting critical smart agricultural components.
(Kim et al., 2019) [72],	Unmanned Aerial Vehicles in Agriculture: A Review of Perspective of Platform, Control, and Applications	UAVs in smart agriculture	The study provides a comprehensive overview of UAVs in smart agriculture, highlighting the latest applications and trends, key technologies, and equipment used.
(Goel and Bindal, 2018) [73],	Wireless Sensor Network in Precision Agriculture: A Survey Report	Precision agriculture	These researchers have provided a review of wireless sensor networks in smart agriculture, the main sensors used, and their structure.
(Krishna Kagita et al., 2022) [75],	Survey on AI-based IoT and drone-equipped smart agriculture	UAVs in smart agriculture	The authors have surveyed the role of AI-assisted UAVs in smart agriculture.
(Maduranga and Abeysekera, 2020) [80],	Machine learning applications in IoT based agriculture and smart farming: a review	Smart agriculture and integration of AI (especially ML)	A brief overview of ML applications in smart agriculture is provided, categorizing applications into several sections such as plant management, soil management, and so on.
(Kumar et al., 2021) [84],	A Bibliometric Analysis of Plant Disease Classification with Artificial Intelligence using Convolutional Neural Network	Plant disease monitoring in agriculture	The authors have performed a bibliographic analysis of using AI for plant disease detection.
(Bolfe et al., 2020) [87],	Precision and Digital Agriculture: Adoption of Technologies and Perception of Brazilian Farmers	Smart agriculture	The authors have presented the results of a survey they performed using farmers in Brazil about adopting digital technology in agriculture, providing their perspectives on that.
(Ramesh and Rakesh, 2020) [88],	Application of big data analytics and artificial intelligence in agronomic research	AI in smart agriculture	The importance of big data analytics and AI is explained in the study. The authors have further discussed each application scenario that employs big data and AI.
(Hassan et al., 2020) [94],	Technology in precision viticulture: a state-of-the-art review	Precision viticulture	The researchers present a state-of-the-art technology in precision viticulture, highlighting key technologies and applications.
(Al-Sammarraie et al., 2021) [96],	New irrigation techniques for precision agriculture: a review	Precision agriculture	The researchers have reviewed the importance of irrigation techniques associated with precision agriculture with their implementation.
(Kolhar and Jagtap, 2021) [98],	Plant trait estimation and classification studies in plant phenotyping using machine vision – A review	Plant phenotyping	The researchers provide an in-depth overview of imaging techniques such as computer vision and their key applications in plant phenotyping. Further, they provide a survey on current computer vision methods for plant trait estimation and classification.
(Martos et al., 2021) [99],	Ensuring Agricultural Sustainability through Remote Sensing in the Era of Agriculture 5.0	Precision agriculture/remote sensing	The researchers have provided an in-depth overview of remote sensing, focusing mainly on its technologies and implementations in the modern agriculture era.
(Moysiadis et al., 2021) [101],	Smart Farming in Europe	Smart agriculture	The researchers have provided an in-depth review of the adoption of smart farming in Europe, highlighted the latest research efforts, and provided an overview of smart farming projects in Europe.
(Ammoniaci et al., 2021) [103],	State of the Art of Monitoring Technologies and Data Processing for Precision Viticulture	Precision viticulture	The review presents a state-of-the-art precision viticulture technology, focusing especially on monitoring tools.
(de Castro et al., 2021) [105],	UAVs for Vegetation Monitoring: Overview and Recent Scientific Contributions	UAVs in smart agriculture	The researchers have provided an in-depth review of the usage of UAVs for vegetation monitoring in the study.
(Maraveas et al., 2022) [107],	Applications of IoT for optimized greenhouse environment and resources management	Smart agriculture	The authors have provided a comprehensive review of IoT solutions for optimized greenhouse environments highlighting key technologies, their implementations, and main limitations.
(Mentsiev et al., 2019) [108],	Automation and IoT for controlling and analysing the growth of crops in agriculture	Smart agriculture	The researchers have offered an overview of how IoT can be used to automate operations in traditional agriculture and highlight its main applications and key challenges.

Based on the review that we conducted, when compiling this summary of contributions, we noted that most of the reviews and surveys only focused on reviewing a specific scientific area pertaining to smart agriculture. As such, the number of papers covering all aspects of smart agriculture is minimal. Thus, an overview of smart agriculture covering all the involved technologies and providing an extensive reference for the latest applications, practices, challenges, and countermeasures are essential for the growth of the domain. Hence, in order to fill this gap, in this study, starting from the history, foundation of smart agriculture, architecture and components, latest applications and practices, hardware and software components used, a summary of the latest contributions, challenges along with countermeasures, and future directions are highlighted. Table 8 provides a comparison of the existing similar work with our study for better understanding. In a nutshell, our study fulfills all of the criteria, defines in the comparison table, which signifies our work among others. Although many similar works exist, our expectation from our work is to become a good reference for researchers and help them with their future work.

**Table 8:** A comparison of related work with our study.

Reference	The architecture of smart agriculture is discussed	A background on IoT in smart agriculture is discussed	Latest applications are discussed	Latest and innovative agricultural practices are discussed	Success stories are presented	The impact of COVID-19 pertaining to smart agriculture is discussed	Challenges along with countermeasures to such challenges are discussed	Future directions are discussed
(Ayaz et al., 2019) [6],	✓	✓	✓	✓	×	×	×	×
(Mekala and Viswanathan, 2017) [7],	×	×	✓	×	×	×	×	×
(Kim et al., 2020) [9],	✓	✓	✓	×	×	×	×	×
(Navarro et al., 2020) [10],	✓	✓	✓	×	×	×	×	×
(Madushanki et al., 2019) [11],	×	×	✓	×	×	×	×	×
(Thilakarathne et al., 2021) [12],	✓	✓	✓	×	×	✓	×	✓
(Salam and Shah, 2019) [58],	×	×	✓	×	×	×	×	×
(Sott et al., 2021) [67],	×	×	✓	×	×	×	×	✓
(Bolfe et al., 2020) [87],	×	×	×	×	✓	×	×	×
(Moysiadis et al., 2021) [101],	✓	✓	✓	×	×	×	×	×
(Maraveas et al., 2022) [107],	✓	✓	✓	×	×	×	×	×
(Mentsiev et al., 2019) [108],	×	✓	×	×	×	×	×	×
Our review	✓	✓	✓	✓	✓	✓	✓	✓

In order to summarize the latest research that has been done, the following Table 9 provides a summary of the latest applications, technological components (in terms of sensors, controllers and networking components we mentioned earlier) and contributions made through such research. Based on our summary, it is evident that most of the latest research is focused on smart monitoring and control. On the other hand, the most commonly used sensors in the reviewed research include temperature, soil moisture, and humidity sensors, with Arduino being the most frequently used controller. For communication, Wi-Fi is the most commonly used for network communication and offloading data to the cloud or remote servers.

**Table 9:** Summarization of applications and technological components used in the recent research.

Reference	Application area/s covered	Sensor/s used	Controller/s used	Network	Contributions
(Gondchawar and Kawitkar, 2016) [3],	Agriculture robotics/irrigation management/soil condition analysis	Temperature sensor, moisture sensor, ultrasonic sensor	Raspberry Pi	Wi-Fi, ZigBee	The researchers have developed a smart farm management system comprising a remotely controlled GPS-based agriculture robot to monitor environmental parameters and secure the farmland from invaders (e.g., birds). Further, they have developed and integrated intelligent smart irrigation solution to the same system as an extension.
(Sushanth and Sujatha, 2018) [5],	Soil condition monitoring, climate monitoring	Temperature sensor, moisture sensor, ultra-sonic sensor, humidity sensor	Arduino	Wi-Fi, 3G, 4G	In this study, the authors have proposed to develop a smart agriculture system using Arduino that features environment monitoring capabilities and identifies the moment of invaders who harm crops; in such case of any event, the system generates an SMS alert so that the farmers will be notified.
(Adidrana and Surantha, 2019) [8],	Hydroponic farming, nutrient condition analysis	pH sensor, TDS sensor, Temperature sensor, moisture sensor	Arduino NodeMCU	Wi-Fi	In terms of hydroponic farming for lettuce, the researchers have proposed a solution to measure pH level, TDS (Total Dissolved Solids), and nutrient temperature in the applied nutrient solution. Further, the researchers have extended their work toward classifying the nutrient conditions using the k-nearest neighbors algorithm.
(Kodali and Sahu, 2016) [23],	Soil condition analysis	Soil moisture sensor	NodeMCU	Wi-Fi	The researchers have presented a low-cost IoT system measuring soil moisture level of plants in the field. It comprised of ESP8266 microcontroller, soil moisture sensors, and a cloud-based Losant platform for offloading gathered data to the cloud. Further, in case of a soil water drop, the system can generate alerts via email to the farmers.
(Rezk et al., 2021) [24],	Climate condition monitoring, crop monitoring	NA	NA	NA	The researchers suggest a smart farming system and a machine learning predictive model for drought and crop productivity prediction for IoT smart agriculture solutions.
(Pranto et al., 2021) [26],	Usage of blockchain for improving food traceability	NA	NA	NA	The researchers have proposed a blockchain-based IoT data acquisition system for agriculture process development, especially for pre-harvest and post-harvest seasons. They have showcased that with the usage of the system, the customers would be able to know the origin and distribution of food products, where farmers could trace everything from where they have stored seeds. The proposed system uses IoT for the acquisition of data and blockchain to provide robust security
(Gao et al., 2020) [37],	Agriculture machinery (in particular UAV)	NA	NA	NA	The researchers have designed an agriculture framework that utilizes UAVs for monitoring pests and plant diseases in the agriculture field
(Payero et al., 2021) [54],	Soil condition monitoring, smart irrigation	Soil moisture sensor	Arduino	2G,3G	The researchers have developed an IoT solution capable of soil moisture monitoring integrated with a cell phone connection. They have made it using low-cost hardware.
(Zougmoré et al., 2018) [56],	Climate monitoring, smart irrigation, and watering	NA	NA	NA	The authors provide an analysis of how the changes in climate and its variability will affect the agricultural harvest, food security, and poverty in the Sub-Saharan Africa region and suggest those can be resolved to some extent with the adoption of smart agriculture.
(Saha et al., 2018) [60],	Crop monitoring, agriculture machinery	Thermal camera, gas sensor	Raspberry Pi	3G,4G	The authors have proposed a UAV solution equipped with ML and other related IoT sensors for monitoring crops in the field.
(Vincent et al., 2019) [64],	Assessing land suitability	pH sensor, soil moisture sensor, electromagnetic sensor	Raspberry Pi	Wi-Fi	The research proposed a decision support system by integrating sensor networks with AI (e.g., neural networks and multi-layer perceptron) for assessing land suitability.
(Saraf and Gawali, 2017) [65],	Crop monitoring	Ultrasonic sensor, light sensor, temperature sensor, air pollution sensor	Arduino	Wi-Fi	The researchers proposed an IoT solution for monitoring crops.
(Alreshidi, 2019) [66],	AI applications in smart agriculture	NA	NA	NA	The researchers have proposed an AI-powered framework for smart agriculture and provided a review of AI applications and their usage in agriculture.
(Garg et al., 2021) [70],	Precision agriculture	NPK sensor, soil moisture sensor	NodeMCU	Wi-Fi	The researchers have developed a multimodal precision agriculture system capable of intelligent fertilizer, smart irrigation, and crop disease and damage prediction.
(Kashyap et al., 2021) [71],	Precision agriculture, smart irrigation	Temperature sensor. humidity sensor, soil moisture sensor	NA	NA	The researchers have proposed a DL neural network method for smart irrigation in precision agriculture and provided an evaluation of their proposed model.
(Chieochan et al., 2017) [76],	Use of renewable energy for smart agriculture	NA	NodeMCU	Wi-Fi	The researchers have developed a smart off-the-grid solar cell system prototype to provide an alternative power supply to a smart mushroom farm.
(Preethi et al., n.d.) [77],	Smart irrigation	pH sensor, temperature sensor, soil moisture sensor, humidity sensor	Raspberry Pi	Wi-Fi	The researchers have developed an intelligent irrigation management system for smart agriculture using a Rasberry pi device as its main controller
(Vanaja et al., 2018) [78],	Soil condition analysis, smart irrigation	Soil moisture sensor	NodeMCU	Wi-Fi	The authors have developed a soil condition monitoring system for smart agriculture using low-cost hardware devices.
(Kalgapure et al., 2021) [79],	Smart irrigation	pH sensor, temperature sensor, soil moisture sensor	Raspberry Pi	Wi-Fi, 2G,3G	The researchers have developed a low-cost intelligent irrigation system based on low-cost sensors and a Raspberry Pi device.
(Jindarat and Wuttidittachotti, 2015) [81],	Smart irrigation	Soil moisture sensor, temperature sensor	ATMEGA328, Raspberry Pi	Wi-Fi	The researchers have developed a smart irrigation solution and provides an evaluation of its implementation.
(Hong et al., 2021) [82],	Water quality monitoring	pH sensor, temperature sensor, turbidity sensor	Arduino UNO	–	The researchers have developed an Arduino-based water quality monitoring system and found the developed system still needs human involvement and is prone to data inaccuracies at times.
(Pratama et al., 2021) [83],	Soil nutrient monitoring	NPK sensor	NodeMCU	Wi-Fi	The researchers have developed a smart IoT solution for measuring soil nutrients using an NPK sensor in citrus plants which sends gathered data to the cloud for further analysis.
(Saleh et al., 2020) [85],	Yield prediction	NA	NA	NA	The authors have used a hybrid neural network algorithm to predict grain yield with agriculture datasets available.
(Chowdhury et al., 2021) [89],	Plant disease detection	NA	NA	NA	In the study, the authors have proposed a DL architecture based on a convolutional neural network for classifying tomato diseases by monitoring the leaf images.
(Rahadiyan et al., 2022) [90],	Soil nutrient monitoring	Camera sensor	Raspberry Pi	Wi-Fi	The authors have developed an architecture for a hydroponic-based AI-powered (powered by computer vision) solution for identifying micronutrient deficiencies in chili plants.
(Alves et al., 2019) [91],	Digital twin in smart agriculture	Temperature and humidity sensor, soil moisture senor	Raspberry Pi	Wi-Fi	The researchers have created a digital twin in smart agriculture to mimic the behavior of a smart water management platform so that farmers can understand the state of their farms.
(Verdouw et al., 2021) [92],	Digital twin in smart agriculture	NA	NA	NA	The researchers have analyzed how digital twins can advance smart agriculture, explained key concepts, topologies, and their implementations, and proposed a conceptual framework for designing and implementing digital twins in smart agriculture.
(Clohessy et al., 2021) [93],	Plant disease diagnosis	NA	NA	NA	The researchers have developed a plant disease severity assessment tool based on ML image analysis integrated with geolocation.
(Hassan et al., 2020) [95],	Smart irrigation	pH sensor, temperature sensor, soil moisture sensor	NodeMCU	2G,3G	The researchers have developed a smart irrigation system integrating an agriculture robot that can be controlled wirelessly.
(Fisher et al., 2018) [97],	Soil condition analysis	Temperature sensor, soil moisture sensor	Arduino	Wi-Fi	In the study, the researchers have developed an IoT solution to collect a variety of parameters from the field (temperature, soil moisture) and send the gathered data to a monitoring platform based on the cloud (ThingSpeak).
(Mhaisen et al., 2018) [100],	Smart irrigation	Water flow sensor	Arduino	Wi-Fi	The authors present a self-powered IoT monitoring system integrated with the cloud and a mobile app for remotely controlling the system.
(Kour et al., 2022) [102],	Precision agriculture	pH sensor, turbidity sensor	NodeMCU, Arduino	Wi-Fi	Saffron, being one of the most expensive crops, the researchers have proposed a hydroponic-based farming solution for Saffronsaffron cultivation using the NFT (nutrient film technique).
(Zhao et al., 2021) [104],	Plant disease diagnosis	NA	NA	NA	The researchers have proposed a DL-based method (convolutional neural network) for effectively identifying tomato leaf diseases.
(Charumathi et al., 2017) [106],	Smart agriculture (in particular hydroponic farming)	pH sensor, temperature sensor, soil moisture sensor	Arduino	Wi-Fi	The authors have proposed and evaluated the design of a hydroponic farming system that can allow farmers to grow their crops hydroponically.

## 8 Present status, challenges, and countermeasures

Smart agriculture offers profound economic and social implications, with the potential to significantly reduce operational costs and increase profitability. By optimizing resource usage, minimizing waste, and enhancing crop yields, it leverages technologies like IoT, cloud computing, and AI to lower reliance on labor while reducing inputs such as water, fertilizers, and pesticides. Despite these benefits, the high initial investment required for advanced technologies such as drones, sensors, and automation equipment poses a significant barrier, particularly for small-scale farmers in developing regions. Addressing this issue requires governments and private-sector partnerships to provide subsidies or financial assistance, encouraging broader adoption of smart agriculture technologies.

The rise of agritech industries has also created new economic opportunities, establishing markets for IoT devices, AI-powered analytics platforms, and precision farming tools. This growth has catalyzed the emergence of startups and fostered collaborations between technology companies and the agricultural sector, driving innovation and generating employment opportunities.

On a societal level, the adoption of smart agriculture brings significant benefits. Enhanced productivity and sustainability contribute to improved food security, particularly in regions grappling with climate challenges or resource scarcity. Automation and real-time monitoring reduce the physical burden on farmers, improving their quality of life and potentially making agriculture a more attractive career choice for younger generations.

During a time when the world faced the challenges of a global pandemic, the importance of smart agriculture was recognized by every nation more than ever before, owing to its significant economic benefits. Smart agriculture has the capability to transform every aspect of traditional agriculture with the aid of IoT, making smart agriculture technologies available to everyone. With the literature we have reviewed, it is evident that smart agriculture has the potential to revolutionize modern agriculture, balancing the trade-off between environment and agriculture towards a sustainable environment and providing solutions for many challenges pertaining to traditional agriculture, including land preparation, irrigation, yield optimization, drought response, pest and disease management.

By 2023, the patterns and behaviors of global agriculture had significantly evolved, influenced by the challenges and disruptions experienced during the COVID-19 pandemic. Health concerns, labor shortages, and supply chain disruptions posed ongoing challenges for the industry whereas, increased awareness of food security led to a surge in domestic food production through urban farming. To address labor shortages and health concerns, greater adoption of automation, as well as remote monitoring and control methods, became essential. Supply chain resilience was enhanced through the use of tracking sensors, unmanned machinery, and real-time monitoring technologies powered by IoT. Governments must continue to strengthen policies that encourage technology adoption in agriculture and provide funding and incentives to support farmers and agricultural businesses in adapting to these advancements.

Taiwan [19], is one such country that has significantly expanded the adoption of smart agriculture practices in the post-pandemic era, spearheaded by the Taiwan Agriculture Research Institute. Innovative applications, including smart technologies for remote monitoring and control, automation through robotics, AI-driven agricultural digital twins for smarter and timely decision-making, and the promotion of domestic urban agriculture through greenhouses and vertical farming, are paving the way for resilience in the agriculture sector. Furthermore, government initiatives to promote the digitalization of agricultural operations, enhance the efficient use of natural resources, and address the declining availability of migrant labor are expected to drive the adoption of smart agriculture throughout this decade.

Many organizations are currently engaged in designing smart agricultural solutions, and many startup companies are being established to expand the smart agriculture market and reach out to more farmers. As we have seen, most of these solutions (in terms of software and hardware we reviewed) are available on a subscription basis and as cloud-based solutions, but trial versions with limited features are also available, with farmers required to pay more for this using additional features. On the other hand, while there are many commercial solutions available, many people are unaware of free and open-source (when any software is open-source, it grants users the rights to use, study, change, and distribute the software and its source code to anyone and for any purpose) smart agricultural solutions. As a result, it is critical that farmers are aware of these solutions because that will allow them to use technology for free rather than investing higher upfront costs for everything, such as buying IoT solutions from the market. According to our analysis, free and open-source smart agricultural solutions are still growing in the market and have a long way to go before competing with commercial solutions. Nonetheless, most free and open-source solutions are aided by a community of developers and other relevant stakeholders and farmers, which would empower the growth of open-source solutions as any development issues or doubts about technology integration can be easily addressed with the help of these communities which is one of the main reasons behind the success of Israel.

### 8.1 Challenges concerning smart agriculture

Even though IoT has become a major game-changer, being the next big revolution in many industries, there are a lot of challenges associated with adopting this technology. There are a lot of hindrances that prevent the proper adoption of IoT in agriculture, such as the limited communication facilities and harsh environmental conditions [2,10,12]. Technological facilities and infrastructure are worst in rural areas and most developing countries, which prevents them from successfully adopting smarter technologies [2]. This has always been the case for most countries which always hinders their economic development [4], despite their economies' total reliance on agricultural food production and export [2]. Hence in this regard, we intend to discuss the key challenges faced by the industry in the following for a better understanding of our audience.

• Privacy and security

Several smart devices are linked to the Internet in a typical smart agricultural setting for intercommunication and data transmission to the cloud or remote servers [[1], [2], [3]]. This connection to the Internet may lead to adverse consequences, if such hardware, software, or network facilities encompass vulnerabilities and the person who uses such devices are not vigilant enough [52]. Once an IoT device is connected to the Internet, it always brings the device one step closer to getting attacked as malicious users are always in search of these simple vulnerabilities that exist on IoT, through the Internet. Thus, potential security attacks on the pervasive smart agriculture system may lead to disastrous consequences such as network interruptions, interrupting device operations (through denial-of-service attacks) [30], and compromising confidential data [4]. Farmers may not possess adequate knowledge about information security and often they are equipped with basic working knowledge for carrying out day-to-day smart monitoring and controlling work, and this would make a perfect gap for attackers to intrude into smart agricultural systems [4,12,52]. Most often, the smart IoT sensors are deployed in the farming field, where sensor nodes can be stolen and tampered by malicious users, eventually leading to disastrous privacy and security problems such as leakage of sensor data and network interruptions. Furthermore, most of the time, these devices are left on the outside; the interference that may come from outside environments, such as harsh weather conditions and electromagnetic activities, may affect the functionality of the devices, which may also result in the degradation of security functionalities of IoT sensing devices. In this regard, Xing et al. [4] has conducted an experimental evaluation in an agricultural field to see whether external interference may affect the functionality of sensing nodes or not, in their study and explained how it impacts the security and privacy of smart agriculture.

**Fig. 21:**
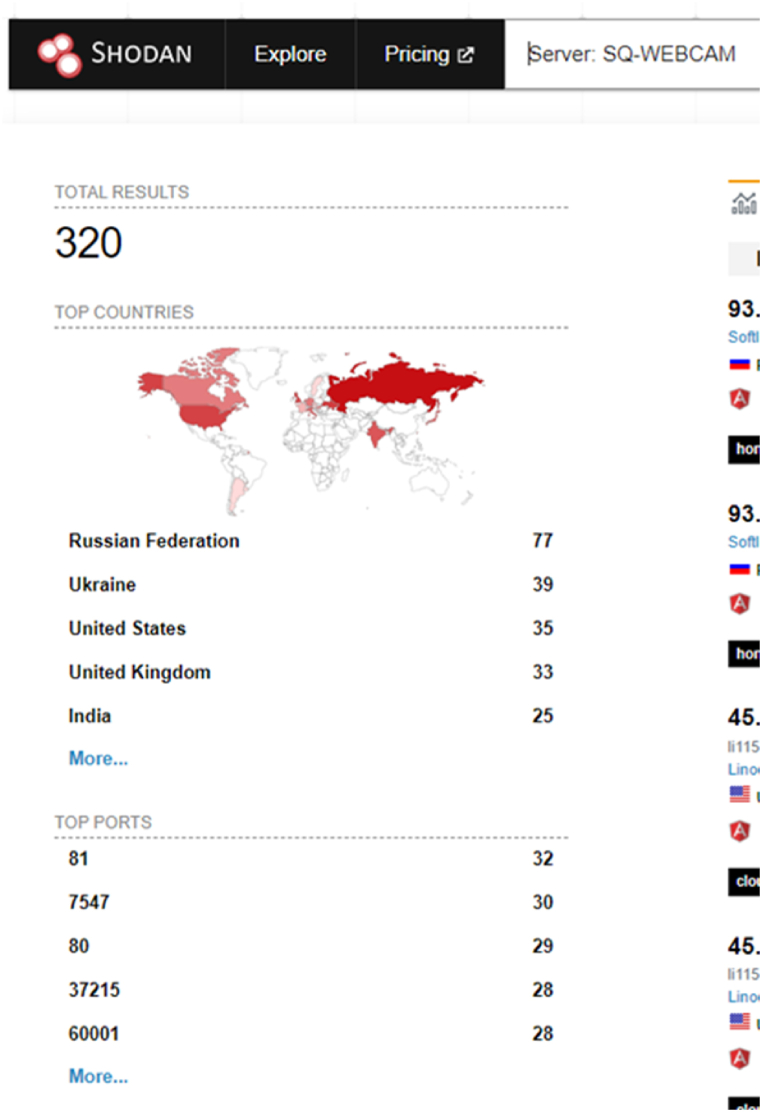
A simple Shodan query for finding vulnerable IP cameras, and webcams worldwide (results can be filtered according to country, vulnerable, open services, and organization).

The ever-increasing cyber threats landscape and simplicity of hacking tools have paved a convenient and super easy way for many malicious users to compromise vulnerable devices through the Internet, which always leads to the growth of cyber-attacks targeting various industries, including smart agriculture. The simplicity of using Google advanced search queries, or Google Dorks, has provided a super-easy way for attackers to target vulnerable devices connected to the Internet. Shodan, a search engine that lets users search various types of digital devices connected to the Internet, provides a good foundation for malicious users to target this vulnerable IoT infrastructure super easily and get to know all available vulnerabilities in the form of a comprehensive report sorted according to various criteria as shown in Fig. 21 [4,52]. Recently, an Israel-based security firm spotted a vulnerability in a smart irrigation system, where around 100 systems were exposed to the Internet, allowing anyone to meddle with the settings [109], which clearly showcased the magnitude of the threat.

In order to properly secure a smart agriculture environment, research should be conducted to strengthen the IoT security and infrastructure security and make sound security solutions that prevent attackers from peeping into smart agricultural networks, and tamper-resistant and anti-interference design must be an integral part of these solutions to make them more securely perfect [52]. On the other hand, as key stakeholders, farmers' awareness should also be increased regarding these security and privacy issues. According to the most recent research [3,4,6,7,12,[30], [31], [32], [33]]. [37,52,68], the following infrastructure and devices used in smart agriculture have more susceptible points to which malicious attackers can gain access.

⁃ UAVs
⁃ Soil, air, crop, and other biosensors
⁃ Wireless access points and wireless connections
⁃ Vertical farming
⁃ Greenhouse
⁃ Radio Frequency Identification (RFID)
⁃ Mobile devices (e.g., desktops, servers, GPS trackers, laptops, mobile devices)
⁃ Cloud computing
• Scalability and reliability

In order to make precise decisions at the right time, precision agriculture requires a significant amount of data processing requiring a large amount of data, which should be collected from underlying IoT sensing nodes [3,12]. This may also require immense support from underlying networking infrastructure, protocols, and undying middleware to offer a smooth and seamless operation. In this regard, this may require highly dependable and reliable hardware and network applications to carry out a heavy workload, pushing these applications to the limits when the data is sent to the fullest capacity. Hence, smart agriculture networks and the remaining infrastructure should always be designed in such a way to guarantee scalability and reliability [12].

• Harsh environmental conditions

In most cases, IoT sensing devices are deployed in open outdoor areas other than indoor controlled environments such as greenhouses [9]. Often these devices deployed in the outdoor environment are exposed to various harsh environmental conditions such as high temperature, heavy rains, strong winds, and humidity, which can affect the overall device operations such as the performance of devices, communication capabilities, and depletion of device power. As a result, IoT devices, networking devices, and infrastructure should be designed to withstand these harsh environmental conditions. In this regard, Zougmoré et al. [56] and Branca et al. [57] have analyzed how the changes in climate and its variability will affect the agriculture harvest, food security, and poverty in Sub-Saharan Africa and suggest how smart technologies can be used to overcome such challenges.

• Perception and knowledge towards adoption of technology

Farmers' knowledge and attitudes toward adopting novel technologies may have a negative impact on the adoption of smart agriculture practices, as some farmers are hesitant to deal with novel technologies and prefer to stick with traditional farming techniques due to a lack of knowledge or their negative attitude toward dealing with new technology [3,6,12]. On the other hand, low perception of people towards farming as a live hood is also becoming common nowadays, especially in developing countries such as Sri Lanka and India, owing to their attitude and caste problems as people in these countries consider that farming is something that only the poor do, which will eventually lead to a shortage of labor for agriculture [12].

• Higher investments

Introducing smarter technologies into agriculture would come at a high cost as the initial configuration and deployment costs are high, including the operation cost for running smart farming services once they are configured. In terms of initial configuration cost, it would come from the costs of the IoT devices, infrastructure and networking equipment, other services (e.g., consultation and cloud services), and labor cost. Operational costs include all costs incurred from running the full smart agriculture services [12]. From our point of view, it can rather be considered an investment, as farmers can reap many benefits as opposed to traditional agriculture with the adoption of smart agriculture. Hence, the government should provide subsidies and funding for farmers to promote smart agriculture nationwide [63].

• Shrinking arable land and scarcity of water resources

The increasing world population expedites the urbanization process, where many people move toward cities in search of prospects. As a result, the city would get bigger and bigger, resulting in a shrinking of arable land and the depletion of water resources that could be used for agriculture. As a side effect, environmental and water pollution may also badly affect agricultural food production, posing a doubt on food security [3,6,9,12]. With the adoption of innovative smart agriculture practices such as greenhouse farming, vertical farming, and other soil-less farming practices, new opportunities for overcoming such challenges associated with the shrinking of arable land, and depletion of water resources are becoming a reality. Most Middle East countries have faced this challenge, and now they have overcome most of these challenges by moving to smart farming with farming in controlled environments [[100], [101], [102], [103], [104]].

Having provided a brief overview of key challenges pertaining to IoT-enabled smart agriculture in the following Table 10 summarize what countermeasures can be taken against overcoming such challenges.

**Table 10:** Summary of countermeasures.

Challenge	Countermeasures
Privacy and security	• Improve the awareness of relevant stakeholders. • Integrate tamper-resistant and anti-interference design with such smart agricultural solutions. • Follow best practices (e.g.: regularly updating software, use of strong username and password combinations) • Implement security measures (e.g.: access control, data encryption, data minimization, data anonymization) • Frequent data auditing • Continuous monitoring and reporting with security solutions • Implement physical security measures (e.g.: real-time surveillance)
Scalability and reliability	• Develop infrastructure in such a way, as to guarantee scalability and reliability • Data minimization • Enable fog, edge data processing
Harsh environmental conditions	• Develop solutions in such a way, as to withstand these harsh conditions • Integrate self-powered IoT with such solutions
Perception and knowledge towards adoption of technology	• Improve the awareness of relevant stakeholders (e.g.: farmers, governments) • Government initiatives towards promoting agriculture (e.g.: providing incentives, integrating necessary subjects into the school curriculum)
Higher investments	• Government should provide subsidies and funding for farmers
Shrinking arable land and scarcity of water resources	• Adoption of innovative smart agriculture practices such as greenhouse farming, vertical farming, and other soil-less farming practices. • Improve the awareness of relevant stakeholders towards promoting urban farming

## 9 The way forward

The 2030 Agenda set out by the United Nations (UN) aims to eradicate world hunger by 2030, whereas with the latest report released by the World Health Organization (WHO) indicates that more than 800 million people worldwide are suffering from hunger, which is nearly one out of every nine people [6,7]. The convergence of IoT and other smart enabling technologies with agriculture already paved the way towards addressing this problem, and so far, we are in the middle of it, as there is a lot more work to be done. Day by day, technologies are evolving, and infrastructure facilities are being developed, which would have a positive impact on the domain, of smart agriculture. Further, many start-up companies are also being set up to introduce novel innovations to the market. Overall, with all these efforts, fully automated smart farming would soon become a reality, which would pave the way for a better future for everyone. The following highlights the anticipated future directions of smart agriculture for better understanding.

• Integration of enabling technologies

Smart agriculture is a collation of many smart enabling technologies backed by IoT. These enabling technologies include communication technologies such as 4G, 5G, 6G, AI, big data, cloud computing, fog computing, edge computing, Network Function Virtualization (NFV), Software-Defined Networking (SDN), and blockchain, which empower smart agriculture to become a promising technology which billions of people can rely on [3,6,12,19,33]. Earlier, agriculture was considered to be a labor-intensive task where resources were very hard to find for integrating with technologies, owing to the power and communication limitations (e.g., Wi-Fi facility in the agricultural field), which can always be backed by these enabling technologies [[37], [38], [39], [40]]. Recent advancements in wireless and cellular communication technologies, such as 5G and 6G, require less power and offer wide communication, over a large area with a lower cost of infrastructure and operational costs [4], presenting opportunities for the expansion of smart agriculture even in rural areas. It is believed that these changes will increase the popularity and utilization of smart agriculture even in rural areas [9]. Additionally, fog and edge computing provide convenient data offloading facilities to the cloud, where they will reduce communication delays when offloading gathered IoT agricultural data to the cloud [4]. Meanwhile, AI is becoming an increasingly important aspect of smart agriculture as being a key technology in precision agriculture, assisting in calculations and then forecasting future status based on previous data or grouping data into classes depending on their real nature. By competing with the ever-increasing demand for agricultural food production, this combined portfolio of technologies offers innovative solutions to improve the harvest, the quality, and productivity of agricultural goods, making smart agriculture a promising technology.

• Efficient large-scale farming

It is evident that smart agriculture in various countries such as Taiwan and Korea have been carried out separately for each specific agriculture process, such as land preparation, sowing, seeding, overall management of plants, harvesting, storage, and distribution [3,[6], [7], [8], [9], [10]]. However, the growing IoT solutions and the IoT-powered management information systems and decision support systems will enable the whole agriculture cycle to be integrated and managed into a more efficient form, where it will further allow for the overall management of entire agriculture processes in one place: using one platform, with super ease. Such a solution would be highly useful for large-scale farming, and currently, further research is carried out towards incorporating smart agriculture into the entire agriculture cycle [[33], [34], [35], [36], [37],^122^].

• Engagement of youth

Engagement of youth or the younger generation, in agriculture is considered essential for the industry's sustainable development [12]. As the world population increases, it will lead to an increase in urbanization which will ultimately result in a shrinking of the rural population and the aging of the rural population [6,7]. This will eventually result in population imbalance and generation shift creating serious problems where the young generation needs to step up to take responsibility. This has always been the case for most developing countries, where around 85 % of young people live in such countries, where agriculture is likely to be their main source of income [12]. Hence it is essential that the younger generation engage more with agriculture, as they can easily comprehend things that deal with technologies compared to the elderly generation.

Conversely, young people will also play an active role in protecting the environment and implementing innovative agricultural solutions, which would be ideal for addressing the labour shortage. As such, it is essential that young people actively participate in agriculture. Governments and policymakers need to take appropriate actions, such as introducing subjects to the curriculum, having awareness and knowledge transfer sessions, and with funding for such initiatives [6,7]. According to Refs. [56,57], with over 200 million population, sub-Saharan Africa has the world's youngest population, where youth unemployment rates are immensely high. Hence, the authors suggested that engaging more young people in the agricultural sector would be an ideal solution for addressing unemployment in the region.

• Growth of unmanned agriculture machinery

Agriculture machinery is an essential item for the expansion of smart agriculture where this machinery is used in every step of smart agriculture, from land preparation up to harvesting [7,12,19,21,37]. In recent years, there has been a significant development in UAVs, and it has contributed immense benefits to agriculture by operating autonomously or piloted remotely without needing a pilot [19,21]. Additionally, there has been a progressive development in the field of other agriculture machinery used for field operations [19,21]. Sooner these in-field machineries are equipped with remote control and autonomous driving facilities, which would increase the productivity of large-scale farms [6,7,9]. Recently with the establishment of 5G technology in Korea, some smart agriculture machinery companies are conducting research on developing self-driving tractors based on IoT and 5G technologies to commercialize them as a product in Korea. Thus, self-driving agricultural vehicles will soon be a reality like UAVs and self-driving electric cars (e.g., Tesla) [9].

• Plant biotechnology

Apart from the soilless farming methods, we have aforementioned (hydroponics, Aquaponics, etc.); there are a few advanced methods that can improve plant capabilities, which the researchers are currently working on, such as phenotyping which is aided by advanced communication, sensing and AI technologies [6,7,9,12,98]. Phenotyping links plant genomics with its ecophysiology and agronomy in precisely controlled lab environments. With the integration of gene sequencing, AI and big data collected from plant phenomics will result in predicting the plant's anatomical, ontogenetical, physiological, and biochemical properties for developing a desirable and favorable plant [6,7,98,[116], [117], [118]]. In this regard, Kolhar & Jagtap [98], provides an in-depth overview of imaging techniques such as computer vision and their key applications in plant phenotyping and survey current machine vision methods for plant trait estimation and classification.

• Blockchain for security and traceability

In most cases, pre-harvest and post-harvest processing are often done using standard farming techniques while tracing and storing harvested data [26]. As a result, farmers aren't getting paid what they're worth, and customers aren't getting adequate information before purchasing their goods, whereas middlemen/processors are raising retail costs. With the integration of blockchain with smart agriculture, these can be totally prevented as it can automate the process while building absolute trust among stakeholders using blockchain based smart contracts, and IoT. On the other hand, with the data gathered from IoT, farmers can now collect data about their crops (nutritional value, other vital bio data and supply chain details) which can then be stored on blockchain [12]. Pranto et al. [26] analyzed the integration of smart contracts and blockchain with IoT devices in pre-harvesting and post-harvesting times of agriculture and proposed an innovative solution that uses blockchain as the backbone where IoT devices collect data from the agriculture field and, smart contracts regulate the interaction among all these contributing parties, enhancing the security and trust of all transactions.

• Adoption of renewable energy and Green IoT

With the ongoing global energy crisis, the study of integrating renewable energy in all aspects has received a lot of interest. For the time being, renewable energy has been widely used in a variety of fields, with agriculture being one of the most promising. Further energy management is critical in smart multi-microgrids for agriculture, and it may secure the energy supply of sensors installed outdoors using wireless charging technology [4,7,[61], [62], [63], [64], [65]]. In this regard, Kjellby et al. [86] developed a prototype of a self-powered IoT device for use in aquaponics and precision agriculture capable of harvesting ambient energy. Nevertheless, reducing the overall energy consumption and keeping the environment safe and clean have also been emphasized with Green IoT, which many smart farming solutions are now looking forward to adopt, as it leads to sustainable smart agriculture, keeping the environment safe with no pollution [[10], [11], [12], [13], [14]].

• Adoption of Virtual Reality (VR) and Augmented Reality (AR)

VR is a virtual reality experience that can be both comparable to and unlike the actual world, whilst AR is a technique for superimposing virtual things onto the real world [4]. Although VR has been widely used as an instructional (for education purposes) tool in the industry, there have been only a few studies in the agricultural field [[4], [5], [6], [7]]. Farmers, for example, can use VR and AR equipment in the control center to control agricultural robots remotely and precisely and use a VR instructional simulator to learn about crop conditions and disease diagnosis [4]. For instance, Kim et al. [110] developed an educational simulator using VR technology for farmer education. Furthermore, there are virtual computer games such as Farming Simulator, which facilitate players to farm, breed livestock, grow crops, and sell assets created from farming, same as in a real environment which would be ideal for farmer training in this smart era.

• Digital twins in agriculture

A Digital Twin is a digital replica of a real-life object that replicates its behaviour and states in a virtual space throughout its lifetime. The use of digital twins as a fundamental tool for farm management allows physical flows to be decoupled from their planning and control [12,91,92]. As a result, farmers can manage operations remotely using real-time digital information instead of relying on direct observation and manual duties on-site. Thus, digital twins hold a lot of promise for bringing smart farming to new levels of production and sustainability, improving overall processes. This enables farmers to act quickly in the presence of any anomalies and allows for modeling the impact of actions using real-world data. Verdouw et al. [92] analyzed how digital twins can be used in smart agriculture in their study and proposed a conceptual framework for designing and implementing digital twins in smart agriculture and provided a comprehensive evaluation of it.

## 10 Conclusion

Based on the review and evaluation we have done, it is evident that IoT enabled smart agriculture has become a prominent domain, which many academics and researchers are currently keen on working on. The bulk of the studies we looked at focused on employing IoT-enabled smart control and monitoring, while the others experimented with a range of approaches to improve the harvest. Despite the fact that IoT is the core of smart agriculture, many hindrances prevent the proper adoption of IoT in smart agriculture, which the current research should focus on. On the other hand, the vital thing is that IoT, the main backbone of smart agriculture, is still in its infancy age, and it would take more time to become a sustainable technology with the current rate of development. IoT is still in its infancy age, possesses a variety of vulnerabilities, and confronts various challenges. Once IoT is converged with agriculture, these vulnerabilities and challenges will also be inherited by the agriculture domain, which should be solved in the long run. The COVID-19 global pandemic placed immense strain on many industries, including the smart agriculture domain. However, it also served as a catalyst for growth in smart agriculture, prompting countries like Taiwan and India to urgently adopt innovative practices to enhance economic benefits and resilience.

The current state of agriculture production urges efficient and smart crop-growing ways to meet the current demand for billions of people, which is subjected to shrinking arable land, decreasing water sources, and environmental pollution, where advanced agricultural practices inside controlled environments have been introduced which uses fewer resources, resulting in better crop yield compared to traditional agriculture. This has now been practiced by many resource-less countries, such as most of the Middle East countries, where most of the land is covered by desert. Soon with the development of technology and with time, IoT and all enabling technologies would be consolidated with all agriculture processes from beginning to end, resulting in higher throughput and less wastage. It will also enable large-scale farming as smart technologies are already being applied and deployed successfully in small-scale farms. Thus, countries with vast areas of barren land can convert these areas into farmlands by adopting smart agriculture, generating more income for the economy, and ensuring food security. On the other hand, it is too early to predict the next big revolution or how far the technologies will go in terms of smart agriculture. However, in our view, we believe that proper and timely adoption of smart agriculture would contribute a lot towards eradicating world hunger; if the UN, international communities, and individual nations can provide adequate and timely support for the adoption of technology. Farmers must comprehend new technology and implement appropriate farming methods to carry out highly efficient agricultural operations that will result in a sustainable crop yield. Considering all these aspects and for the growth of the industry and providence guidance for future researchers, in the study, we have synthesized the current knowledge of smart agriculture, highlighting its layered architecture and description of each of the layers, key application areas in terms of their importance and popularity in recent years, advanced and innovative agricultural practices such as greenhouse farming and vertical farming, software and hardware that are currently being utilized, success stories, key challenges that prevent the proper adoption and countermeasures towards tackling them, future directions, and providing a summary on recent contributions, and categorizing them in a systematic way. With this rate, we believe that one day man will be able to colonize other planets such as Mars and planets far away from our solar system and carry out farming in controlled environments with the aid of these smarter technologies making all science fiction a reality.

## CRediT authorship contribution statement

**Navod Neranjan Thilakarathne:** Writing – original draft, Visualization, Validation, Software, Resources, Project administration, Methodology, Investigation, Formal analysis, Data curation, Conceptualization. **Muhammad Saifullah Abu Bakar:** Writing – review & editing, Supervision, Resources, Investigation. **Pg Emeroylariffion Abas:** Writing – review & editing, Supervision, Project administration. **Hayati Yassin:** Writing – review & editing, Supervision, Methodology, Funding acquisition.

## Data availability

No new data was generated for the research described in the article.

## Declaration of competing interest

The authors declare that they have no known competing financial interests or personal relationships that could have appeared to influence the work reported in this paper.
